# Oxidative stress marker aberrations in children with autism spectrum disorder: a systematic review and meta-analysis of 87 studies (*N* = 9109)

**DOI:** 10.1038/s41398-020-01135-3

**Published:** 2021-01-05

**Authors:** Lei Chen, Xiao-Jie Shi, Hua Liu, Xiao Mao, Lue-Ning Gui, Hua Wang, Yong Cheng

**Affiliations:** 1 Key Laboratory of Ethnomedicine of Ministry of Education, Center on Translational Neuroscience, College of Life and Environmental Sciences, Minzu University of China, Beijing, China; 2 NHC Key Laboratory of Birth Defects Research, Prevention and Treatment (Hunan Provincial Maternal and Child Health Care Hospital), Hunan, China

**Keywords:** Autism spectrum disorders, Molecular neuroscience

## Abstract

There is increasing awareness that oxidative stress may be implicated in the pathophysiology of autism spectrum disorder (ASD). Here we aimed to investigate blood oxidative stress marker profile in ASD children by a meta-analysis. Two independent investigators systematically searched Web of Science, PubMed, and Cochrane Library and extracted data from 87 studies with 4928 ASD children and 4181 healthy control (HC) children. The meta-analysis showed that blood concentrations of oxidative glutathione (GSSG), malondialdehyde, homocysteine, *S*-adenosylhomocysteine, nitric oxide, and copper were higher in children with ASD than that of HC children. In contrast, blood reduced glutathione (GSH), total glutathione (tGSH), GSH/GSSG, tGSH/GSSG, methionine, cysteine, vitamin B9, vitamin D, vitamin B12, vitamin E, *S*-adenosylmethionine/*S*-adenosylhomocysteine, and calcium concentrations were significantly reduced in children with ASD relative to HC children. However, there were no significance differences between ASD children and HC children for the other 17 potential markers. Heterogeneities among studies were found for most markers, and meta-regressions indicated that age and publication year may influence the meta-analysis results. These results therefore clarified blood oxidative stress profile in children with ASD, strengthening clinical evidence of increased oxidative stress implicating in pathogenesis of ASD. Additionally, given the consistent and large effective size, glutathione metabolism biomarkers have the potential to inform early diagnosis of ASD.

## Introduction

Autism spectrum disorder (ASD) is a complicated, pervasive, and heterogeneous disease^1^, which is characterized by varying degrees of dysfunctional social communication, narrow interests, and repetitive behaviors^2,3^. The world-wide prevalence of the disease has been reported to be approximately 1%, with a male-to-female ratio of 4:1^4^. Recently, data from the Centers of Disease Control and Prevention showed that the prevalence of ASD reached 1/54 children in the United States. Although the pathophysiology of ASD is still poorly understood, the interplay between genetic and environmental factors has been suggested to cause the disease^5^. Until now, the effort to search for biomedical treatment on ASD core symptoms is fruitless. However, it is known that early intervention in children with ASD would better improve social skill later in life. Unfortunately, there is no reliable biomarker for early diagnosis of ASD, and it is difficult to diagnose ASD children aged <2 years because core behavioral symptoms can only be tested in older children^6^. Thus there is a need to better elucidate the ASD etiology and find biomarkers for early diagnosis and subsequently develop disease-modifying treatment for ASD.

The social communication problem as the core diagnostic feature in ASD children has been extensively studied in the field. However, the non-diagnostic features in ASD children have attracted great attention over the past decade; these include gastrointestinal problem^7^, sleep disorders^8^, immune system aberration^9^, neurotrophic factor dysregulation^10^, mitochondrial dysfunction^11^, and oxidative stress^12^. Oxidative stress may cause damages to lipids, proteins, and DNAs in cells, with subsequent disease development in humans^13^. The human brain is especially vulnerable to oxidative stress because it accounts for only 2% of body mass but consumes 20% of oxygen^14^. Moreover, results from animal studies have consistently demonstrated the detrimental effects of oxidative stress on central nervous system^15,16^. Therefore, it has been proposed that oxidative stress in the developing brains contributes to neuronal damage in genetically susceptible children, which is important in the pathophysiology of ASD.^6,17^. Indeed, an increased oxidative stress was observed in the brains of ASD patients^18^.

Additionally, studies from different laboratories investigated peripheral oxidative stress marker dysfunctions in ASD patients in recent years; this is of potential importance not only for the understanding of ASD pathogenesis but also for supporting the diagnosis of ASD and monitoring the disease progression. Several reports have shown abnormal oxidant and antioxidant concentrations in blood of children with ASD relative to healthy control (HC) children^19–21^. However, clinical data were inconsistent among studies^22–24^. Therefore, it is necessary to evaluate oxidative stress status in ASD children with a meta-analysis.

Here we systematically searched the literature to identify studies measuring oxidative stress-related biomarkers in ASD patients and controls and pooled the data to improve the strength of increasing evidence implicating oxidative stress in the ASD pathogenesis.

## Method

### Article selection process

This study followed the Preferred Reporting Items for Systematic Reviews and Meta-Analyses statement^25^. Web of Science, PubMed, and Cochrane Library from September 2018 to June 2020 were systematically searched by two investigators, and the published date for searched papers from the databases ranged from June 1990 to June 2020. Only peer-reviewed English articles were included in this study. The following search term was used: (autism or autistic disorder or ASD) and (oxidative stress or glutathione or superoxide dismutase or catalase or malondialdehyde or ceruloplasmin or hydroxy guanosine or transferrin or copper or low density lipoprotein or cholesterol or antioxidants or oxidoreductases or vitamin or carotene or folic or folate or amino acid or methionine or cysteine or homocysteine or cystathionine or cysteinyl glycine or superoxide or TBARS or lipid peroxidation or nitric oxide or iron or ferritin). We included articles if there were serum or plasma oxidative stress marker data on children with ASD and controls. Studies were excluded if: (1) without sufficient data; (2) potential biomarkers were not assessed from human samples; (3) without normal control children; (4) studies used the same cohort of samples; (5) paper was retracted; (6) samples were not from plasma or serum; (7) a single marker was evaluated in <3 studies; (8) samples were from adult patients with ASD.

### Data extraction

We extracted potential biomarker concentrations with standard deviation (SD), sample size, and *P* values for effective size (ES) generation. The information on country (latitude), gender, age, sampling source, publication year, assay type, and diagnosis from the included studies were also recorded (eTable 1). The Newcastle–Ottawa quality assessment scale was performed to assess the qualities of the included studies (eTable 2).

### Data analysis

The Comprehensive Meta-analysis software was utilized to process the data extracted from the included studies. Sample size and oxidative stress marker level with SD were primarily used to evaluate ES. In some cases, we used sample size and *P* value for ES generation due to a lack of data on oxidative stress marker concentration. The Hedge’s *g* statistic (ES) for each oxidative stress marker was calculated as previously described^26^. We chose the random-effects model for the study if there was significant between-study heterogeneity^10^, and the rest were performed by a fixed-effect model. Furthermore, sensitivity analysis^27^, *I*^2^ statistic and Cochrane *Q* test^28^, meta-regressions^29^, and Egger’s test^30^ were performed as previously described. Statistical significance was set at *P* < 0.05 in the present meta-analysis, an exception was the Cochrane *Q* test (*P* < 0.10 was considered statistically significant).

## Results

Initial screenings identified 5935, 3999, and 230 records from Web of Science, PubMed, and Cochrane Library, respectively. These records were screened, which led to full-text scrutiny of 212 articles. After carefully reading the 212 articles, we excluded 125 articles because of the following reasons: no necessary data (*n* = 73), paper was retraced (*n* = 1), markers were analyzed in animal model (*n* = 2), without an HC (n = 24), samples were not from serum or plasma (*n* = 21), samples were from adult patients with ASD (*n* = 4). Therefore, we included a total of 87 articles in this study (eReference), and a flowchart is presented in eFig. 1.

### Oxidative stress-related biomarker dysregulations in ASD

**Fig. 1:**
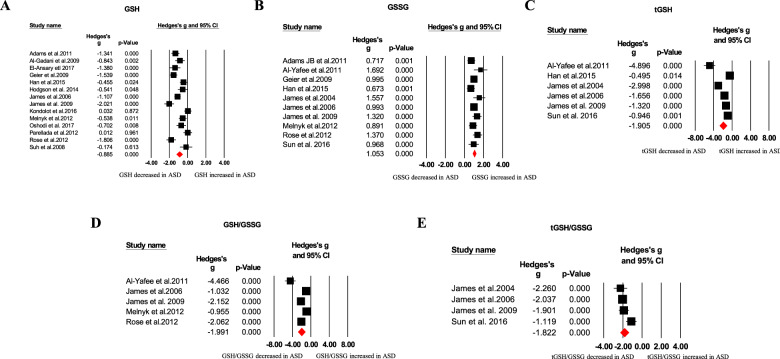
Forest plots of effect sizes for glutathione metabolism. Meta-analysis of pooled data from the included studies showing the association between GSH (**A**), GSSG (**B**), tGSH (**C**), GSH/GSSG (**D**), tGSH/GSSG (**E**), and ASD. GSH reduced glutathione, GSSG oxidative glutathione, tGSH total glutathione, ASD autism spectrum disorder.

**Fig. 2:**
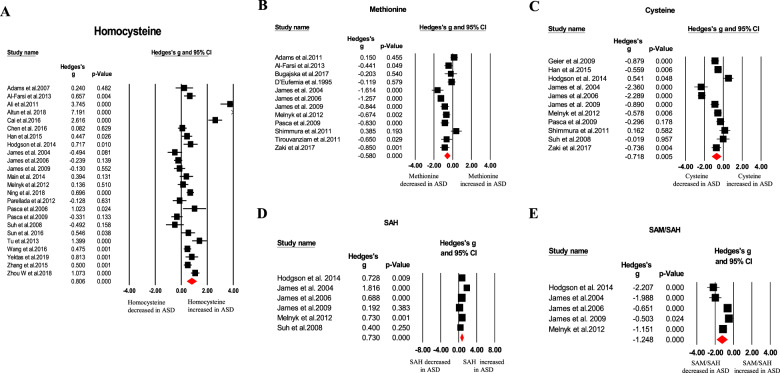
Forest plots of effect sizes for transmethylation cycle and transsulfuration pathways. Meta-analysis of pooled data from the included studies showing the association between homocysteine (**A**), methionine (**B**), cysteine (**C**), SAH (**D**), SAM/SAH (**E**), and ASD. SAH *S*-adenosylhomocysteine, SAM/SAH *S*-adenosylmethionine/*S*-adenosylhomocysteine, ASD autism spectrum disorder.

**Fig. 3:**
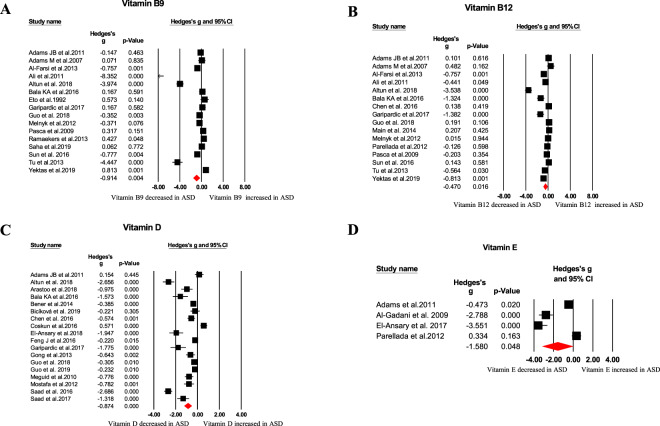
Forest plots of effect sizes for vitamins. Meta-analysis of pooled data from the included studies showing the association between vitamin B9 (**A**), vitamin B12 (**B**), vitamin D (**C**), vitamin E (**D**), and ASD. ASD autism spectrum disorder.

**Fig. 4:**
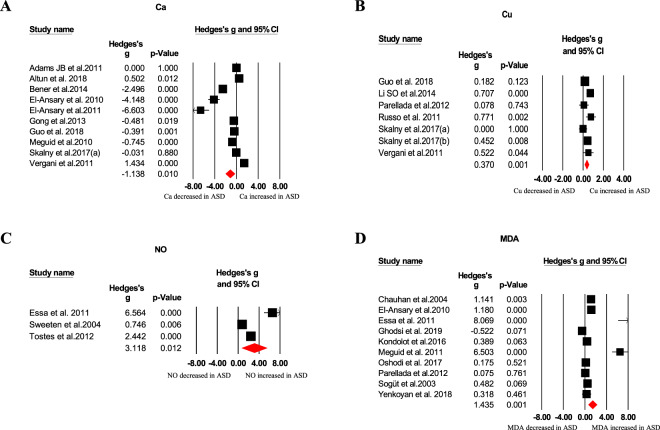
Forest plots of effect sizes for trace elements, NO, and MDA. Meta-analysis of pooled data from the included studies showing the association between Ca (**A**), Cu (**B**), NO (**C**), MDA (**D**), and ASD. Ca calcium, Cu copper, NO nitric oxide, MDA malondialdehyde, ASD autism spectrum disorder.

Our meta-analysis revealed blood oxidative glutathione (GSSG), homocysteine, *S*-adenosylhomocysteine (SAH), copper, nitric oxide, and malondialdehyde (MDA) concentrations were significantly higher in ASD children than that of HC children, as showed in Table 1 and Figs. 1–4. In contrast, we found blood total glutathione (tGSH), reduced glutathione (GSH), GSH/GSSG, tGSH/GSSG, methionine, cysteine, *S*-adenosylmethionine (SAM)/SAH, vitamins (B9, B12, D, and E), and calcium concentrations were significantly decreased in ASD children relative to controls (Table 1 and Figs. 1–4). Furthermore, other markers including glutathione-*S*-transferases (GSH), superoxide dismutase, transferrin, catalase, glutathione peroxidase, vitamin A, vitamin B6, vitamin C, cystathionine, iron, zinc, magnesium, ceruloplasmin, total cholesterol, triglycerides, ferritin, and SAM did not significantly associate with ASD (Table 1).

**Table 1:** Summary of comparative outcomes for measurements of blood biomarker levels.

Marker	No. of studies	No. with ASD/controls	Main effect			Heterogeneity				Publication bias	
			Hedges *g* (95% CI)	*Z* score	*P* value	*Q* statistic	df	*P* value	*I*^2^ statistic	Egger intercept	*P* value
GSH	14	583/624	−0.885 (−1.224 to −0.546)	−5.122	<0.001	97.271	13	<0.001	86.635	−1.32259	0.76091
GSSG	10	410/506	1.053 (0.865 to 1.241)	10.992	<0.001	15.262	9	0.084	41.029	4.22184	0.02392
tGSH	6	239/247	−1.905 (−2.722 to −1.087)	−4.567	<0.001	71.317	5	<0.001	92.989	−7.806495	0.07835
GSH/GSSG	5	218/230	−1.991 (−2.792 to −1.189)	−4.866	<0.001	48.759	4	<0.001	91.796	−8.92767	0.01552
tGSH/GSSG	4	169/177	−1.822 (−2.279 to −1.365)	−7.814	<0.001	9.103	3	0.028	67.046	0.96789	0.86858
Homocysteine	24	1131/1131	0.806 (0.431 to 1.181)	4.214	<0.001	402.835	23	<0.001	94.290	5.34085	0.04937
Methionine	12	473/456	−0.580 (−0.894 to −0.265)	−3.612	<0.001	59.149	11	<.001	81.403	1.28310	0.74076
Cysteine	11	430/442	−0.718 (−1.214 to −0.221)	−2.833	0.005	117.857	10	<0.001	91.515	3.01921	0.62529
*S*-adenosylhomocysteine	6	238/240	0.730 (0.362 to 1.098)	3.888	<0.001	17.530	5	0.004	71.447	2.22492	0.51958
SAM/SAH	5	207/229	−1.248 (−1.839 to −0.658)	−4.144	<0.001	30.823	4	<0.001	87.022	−8.23393	0.04265
Vitamin B9	16	863/596	−0.914 (−1.529 to −0.298)	−2.910	0.004	388.379	15	<0.001	96.138	−6.46188	0.05986
Vitamin B12	16	831/655	−0.470 (−0.853 to −0.087)	−2.404	0.016	177.868	15	<0.001	91.567	−6.3824	0.02396
Vitamin D	18	1803/1472	−0.874 (−1.218 to −0.529)	−4.969	<0.001	342.876	17	<0.001	95.042	−5.56954	0.01683
Vitamin E	4	140/136	−1.580 (−3.144to−0.016)	−1.980	0.048	90.750	3	<0.001	96.694	−14.09468	0.12005
NO	3	72/70	3.118 (0.682 to 5.555)	2.509	0.012	51.5	2	<0.001	96.117	11.02569	0.11033
Ca	10	888/644	−1.138 (−2.007 to −0.269)	−2.568	0.010	446.367	9	<0.001	97.984	0.24791	0.96606
Cu	7	666/354	0.370 (0.154 to 0.586)	3.361	0.001	13.512	6	0.036	55.594	1.87409	0.41493
MDA	10	278/244	1.435 (0.622 to 2.248)	3.458	<0.001	150.596	9	<0.001	94.024	9.03332	0.00385
Glutathione peroxidase	9	233/233	−0.242 (−1.335 to 0.850)	−0.434	0.664	247.381	8	<0.001	96.766	−4.11131	0.56577
Glutathione-*S*-transferases	3	82/63	−0.874 (−1.878 to 0.131)	−1.705	0.088	15.960	2	<0.001	87.469	−15.42681	0.05110
Cystathionine	4	170/160	0.029 (−0.459 to 0.517)	0.117	0.907	12.718	3	0.005	76.412	−3.85774	0.40622
*S*-adenosylmethionine	5	211/213	−0.447 (−0.975 to 0.081)	−1.659	0.097	25.717	4	<0.001	84.446	1.74195	0.74482
Vitamin A	7	245/231	−0.120 (−0.333 to 0.094)	−1.100	0.271	26.329	6	<0.001	77.211	1.74773	0.52178
Vitamin B6	4	205/130	−1.191 (−3.249 to 0.868)	−1.134	0.257	159.088	3	<0.001	98.114	−6.87342	0.66797
Vitamin C	3	113/103	0.033 (−0.456 to 0.521)	0.131	0.896	6.501	2	0.039	69.237	−10.98625	0.30542
SOD	15	497/435	−0.410 (−0.910 to 0.089)	−1.611	0.107	178.604	14	<0.001	92.161	−2.71962	0.38952
Fe	7	610/425	0.161 (−0.312 to 0.634)	0.667	0.505	73.694	6	<0.001	91.858	9.63277	0.01165
Zn	8	741/431	−0.001 (−0.440 to 0.438)	−0.005	0.996	77.743	7	<0.001	90.996	5.59972	0.21712
Mg	6	675/483	−0.183 (−0.446 to 0.081)	−1.360	0.174	17.551	5	0.004	71.512	3.58971	0.01270
Catalase	5	152/150	−0.106 (−0.664 to 0.452)	−0.371	0.711	21.958	4	<0.001	81.784	8.28761	0.00878
Total cholesterol	4	168/170	−0.702 (−2.378 to 0.974)	−0.821	0.412	140.655	3	<0.001	97.867	−7.08445	0.93969
Triglycerides	3	95/97	0.330 (−0.429 to 1.090)	0.852	0.394	13.981	2	0.001	85.695	33.89977	0.07102
Ceruloplasmin	6	153/115	−0.190 (−0.635 to 0.255)	−0.838	0.402	15.667	5	0.007	68.087	3.21102	0.49831
Transferrin	4	112/94	−0.072 (−0.545 to 0.402)	−0.296	0.767	8.777	3	0.032	65.819	−13.81589	0.00157
Ferritin	6	717/548	0.178 (−0.147 to 0.503)	1.072	0.284	31.178	5	<0.001	83.963	5.17107	0.00305

*df* degrees of freedom, *ASD* autism spectrum disorder, *GSH* reduced glutathione, *GSSG* oxidized glutathione, *tGSH* total glutathione, *GSH/GSSG* reduced glutathione/oxidized glutathione ratio, *SAM/SAH* *S*-adenosylmethionine/*S*-adenosylhomocysteine ratio, *NO* nitric oxide, *Ca* calcium, *Cu* copper, *MDA* malondialdehyde, *SOD* superoxide dismutase, *Fe* iron, *Zn* zinc, *Mg* magnesium.

### Investigation of heterogeneity

Our results showed that there was no significant between-study heterogeneity for studies measuring GSSG concentrations, whereas the other 34 oxidative stress-related markers showed significant between-study heterogeneities.

We then examined whether sampling source, sex, age, publication year, latitude, and source of healthy volunteers were variables that could affect the meta-analysis outcome. Subgroup analyses and meta-regressions were performed on homocysteine, methionine, cysteine, vitamin B9, vitamin B12, vitamin D, and GSH because these markers were measured in more than ten studies, and the levels of these markers were dysregulated in children with ASD.

The high levels of heterogeneities were found among studies analyzing plasma (*I*^2^ = 91.964; 10 articles) and serum (*I*^2^ = 94.773; 14 articles) homocysteine, and plasma (*I*^2^ = 62.187, 5 articles) and serum (*I*^2^ = 93.931, 11 articles) vitamin B12 concentrations. Moreover, serum vitamin B12 and homocysteine concentrations were significantly associated with ASD, whereas plasma vitamin B12 and homocysteine concentrations were not statistically different between children with ASD and HC children (eFig. 2). Considering the small number of studies that analyzed serum methionine, serum cysteine, plasma vitamin B9, plasma vitamin D, and serum GSH levels, we therefore performed subgroup analysis on plasma methionine, plasma cysteine, serum vitamin B9, serum vitamin D, and plasma GSH. We found significant differences between children with ASD and control children regarding studies measuring plasma methionine, serum vitamin B9, serum vitamin D, and plasma GSH levels (eFig. 2), and the between-study heterogeneities were unchanged.

Given that age and sex ratio were unevenly distributed between the ASD and HC groups and the HC subjects were from different sources (local community, kindergarten/primary school and clinical center) in some included studies (eTable 1), we performed the stratified analyses based on the three factors. Stratifications based on the three factors showed different between-study heterogeneities for several subgroups (eFig. 2 and eTable 3), suggesting that the source of HC group, unevenly distributed age, and sex ratio were three confounding factors that may affect the meta-analysis outcome.

Meta-regression analyses for homocysteine, cysteine, and vitamin B12 only showed that publication year was significantly associated with ES, as shown in eFig. 3. Additionally, age was a confounding factor on the meta-analysis outcome for studies analyzing vitamin B9 (eFig. 3). For methionine, vitamin D, and GSH, age, sex, publication year, and latitude did not significantly affect the study outcome (eFig. 3).

Sensitivity analysis suggested that the differences on homocysteine, methionine, cysteine, vitamin B9, vitamin B12, vitamin D, and GSH between ASD patients and HC subjects were not influenced by any individual study, indicating the strength of the meta-analysis outcome.

We visually inspected the funnel plots and did not find evidence of publication bias regarding studies measuring blood methionine, cysteine, vitamin B9, and GSH levels, whereas potential publication bias was observed for homocysteine, vitamin B12, and vitamin D (eFig. 4A–G). The absence or presence of publication bias was confirmed through the Egger’s test, as shown in Table 1.

## Discussion

Because of the large number of studies that analyzed blood oxidative stress markers in ASD children in recent years, here we were able to include 87 studies with 4928 ASD children and 4181 controls, and revealed that 18 oxidative stress-related markers were dysregulated in blood (serum or plasma) of children with ASD. These 18 oxidative stress markers included glutathione metabolism, transmethylation cycle and transsulfuration pathways, vitamins, and trace elements. Specifically, our meta-analysis demonstrated that blood GSSG, MDA, homocysteine, SAH, nitric oxide, and copper concentrations were significantly increased in children with ASD children relative to HC subjects. In contrast, levels of blood GSH, tGSH, GSH/GSSG, tGSH/GSSG, SAM/SAH, methionine, cysteine, vitamins (B9, B12, D, and E), and calcium were significantly decreased in ASD children relative to control subjects. Interestingly, previous studies have shown the associations of ASD with polymorphisms in the genes of oxidative stress markers. A very recent study by Yu et al. showed that genetic polymorphisms in vitamin D metabolism-related enzymes were associated with the risk and severity of ASD^31^, and a meta-analysis suggested that vitamin D receptor rs731236 and rs7975232 were significantly associated with ASD^32^. Moreover, Haghiri et al. found that methionine synthase A2756G gene polymorphism (rs1805087) was associated with ASD occurrence in northern Iran^33^. However, methionine synthase rs1805087 was not a risk factor for the susceptibility and disease severity to ASD in a Chinese population^34^. Nevertheless, these results provide strong clinical evidence indicating that children with ASD were accompanied by oxidative stress marker aberrations, and the dysregulated oxidative stress marker profile in ASD children may offer novel insight into molecular pathways that confer vulnerability to the disease onset and/or progression.

Whether increased oxidative stress caused the behavioral problems in ASD children is still under debate. However, the increased oxidative stress that contributed to ASD onset and/or development is reasonable since considerable data suggests beneficial effects of antioxidants in ASD, both from clinical and preclinical studies. Mousavinejad et al. found reduced oxidative stress in ASD children supplemented with Coenzyme Q10, and the supplementation also improved the children’s gastrointestinal problems and sleep disorders^35^. Consistently, carnosine supplementation has been found to be effective in improving sleep problem in ASD children, especially for sleep duration and parasomnias^36^. Another clinical trial supported the usefulness of the antioxidant, *N*-acetylcysteine, for treating irritability in children with ASD^37^. The beneficial effects of *N*-acetylcysteine in ASD was supported by preclinical studies suggesting that it improved repetitive and stereotypic behaviors in autism model rats via its antioxidant properties^38^. Interestingly, studies showed that vitamin D supplementation improved behavioral symptoms in ASD children, including stereotypy, irritability, and eye contact, as evaluated by the Autism Behavior Checklist and/or the Childhood Autism Rating Scale^39–41^. Our present meta-analysis showed reduced vitamin D levels in ASD children and therefore supporting results from clinical trials on vitamin D supplementation in patients with ASD. However, it should be noted that one study failed to find beneficial effects of vitamin D supplementation in patients with ASD, although sample size in this trial is relatively small^42^. Nevertheless, these above results suggested novel therapeutic targets for ameliorating clinical symptom of ASD children.

**Fig. 5:**
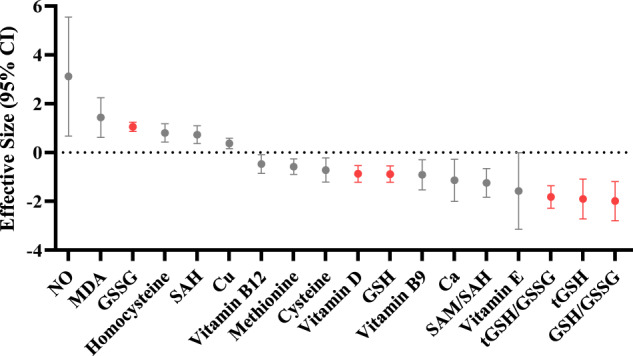
Biomarker performance rating for autism spectrum disorder. Head-to-head biomarker performance in blood oxidative stress markers based on effective size (95% CI). Highlighted oxidative stress markers with red indicate high priority for potential diagnosis of autism spectrum disorder.

In addition to a better understanding of the etiology of ASD, data from the present meta-analysis has implications for the diagnosis of ASD children. Currently, there is no validated biomarker to diagnose or monitor the course of major neuropsychiatric diseases, including depression, schizophrenia, and ASD, and the diagnosis of these diseases rely on clinical symptoms that was made solely by subjective evaluation of clinicians. Identifying biomarker for ASD is particularly important because it is very difficult to perform behavioral tests in ASD children aged <2 years, and ASD children were usually diagnosed long after the disease onset, therefore preventing affected families and society from early intervention in children with ASD. Indeed, great efforts have been made in the field over the past two decades to search objective biomarkers to inform the diagnosis of ASD, especially biomarkers from blood since it is easily accessible and inexpensive. Potential blood biomarkers have been proposed; these include inflammatory cytokines, neurotrophic factors, and miRNAs^10,43,44^. However, these studies have not led to clinically useful biomarker to diagnose ASD, partly due to inconsistent results from different laboratories. In this meta-analysis, we found significant associations between several blood oxidative stress marker levels with ASD indicating that these factors are potential biomarkers for ASD. We further compared the biomarker performance of oxidative stress makers based on ES, and the interesting finding is that the studies that analyzed blood glutathione metabolism markers showed consistent and high significant differences between ASD children and controls, and the potential biomarkers included GSH, tGSH, GSSG, tGSH/GSSG, and GSH/GSSG (Fig. 5). Based on these findings, it is likely that the glutathione metabolism markers had good sensitivity and specificity for the diagnosis of ASD, and future clinical studies should be well designed to validate this hypothesis. Additionally, there is an increasing awareness that the integration of different biomarkers would increase accuracy for the early diagnosis of ASD. Therefore, combining glutathione with other markers that reflects various dysregulated molecular pathways, such as vitamin D, would likely result in a better approach for the early diagnosis of ASD.

We found significant heterogeneities among studies for many analyzed potential biomarkers in ASD. The sampling source (serum or plasma) did not address the heterogeneities, whereas stratifications base on unevenly distributed age and sex ratio between the ASD and HC groups revealed different between-study heterogeneities for several subgroups, indicating potential moderating effects of age and sex on the meta-analysis outcome. Sex had a moderating effect on the meta-analysis outcome and is reasonable since it was known that the male-to-female ratio was 4:1 for ASD^4^. Furthermore, meta-regressions demonstrated that publication year significantly affected the associations between homocysteine, cysteine, and ASD, and the association between vitamin B9 and ASD was affected by age. Therefore, we at least partially addressed the between-study heterogeneities through subgroup and meta-regression analyses, which is a strength for the present meta-analysis.

Notwithstanding its significant strengths, some inherent limitations were present in this meta-analysis. The first limitation is that we demonstrated oxidative stress marker concentration changes in peripheral blood of ASD children, but how much the peripheral measurements reflect oxidative stress activity within the brain is unknown. Cerebrospinal fluid samples from ASD children should be useful to gain better insight into the potential etiological role of oxidative stress in ASD, although it is difficult to obtain cerebrospinal fluid samples from children. However, a postmortem study showed that GSH and GSH/GSSG were significantly decreased in the brains of ASD patients relative to controls^18^, suggesting that markers of glutathione metabolism may have parallel changes between central and peripheral in ASD. Second, the information on medication status and disease severity in the included studies were limited, therefore it is unknown whether these variables affect oxidative stress markers in children with ASD. Third, we found potential publication bias for studies analyzing vitamin B12, vitamin D, and homocysteine, and this may confound findings of the meta-analysis. One potential reason for the observed publication bias is that only English articles were included, which is a limitation for this study. However, since the number of non-English articles on the associations between ASD and oxidative stress markers were very limited, and no evidence of publication bias for most analyzed oxidative stress markers, it is unlikely that this would significantly influence our meta-analysis outcome.

## Conclusions

The results from this study showed that blood GSSG, MDA, homocysteine, SAH, nitric oxide, and copper concentrations were significantly increased, whereas GSH, tGSH, GSH/GSSG, tGSH/GSSG, SAM/SAH, methionine, cysteine, vitamins (B9, B12, D, and E), and calcium concentrations were significantly reduced in children with ASD. Due to the consistent and large ESs for the associations between glutathione metabolism markers, vitamin D, and ASD, they have the potential to be used as diagnostic markers for ASD. Therefore, future investigations into oxidative stress markers as potential early diagnosis and therapeutic target of ASD are necessary.

## Supplementary information

Supplementary combined
