# Supplementary material for: Oxidative stress marker aberrations in children with autism spectrum disorder: a systematic review and meta-analysis of 87 studies (N = 9109)
Source: Transl Psychiatry. 2021 Jan 5;11:15. doi: 10.1038/s41398-020-01135-3 (PMC7791110; doi:10.1038/s41398-020-01135-3)
Supplement: Supplementary file 1 — Supplementary combined [file 41398_2020_1135_MOESM1_ESM.pdf]

Supplementary Online Content

eTable 1: Characteristics of included studies measuring blood oxidative stress markers.

eTable 2: Newcastle-Ottawa quality assessment scale for included studies.

eTable 3. Sub-group analysis stratified by source of healthy volunteer for homocysteine, methionine, vitamin B9, vitamin B12, vitamin D and GSH.

eFigure 1: PRISMA flowchart of the literature search.

eFigure 2: Sub-group analysis stratified by source of sampling, age matched and gender matched for homocysteine, methionine, cysteine, vitamin B9, vitamin B12, vitamin D and GSH.

eFigure 3: Meta-regressions of age, gender, publication year and latitude for homocysteine, methionine, cysteine, vitamin B9, vitamin B12, vitamin D and GSH.

eFigure 4: Funnel plots for homocysteine, methionine, cysteine, vitamin B9, vitamin B12, vitamin D and GSH.

eReference: Included 87 studies for meta-analysis.

eTable 1: Characteristics of included studies measuring blood oxidative stress markers

Study/Year	Markers Measured	Country	Samples (ASD/HC)	Gender (%Male) (ASD/HC)	Mean Age (ASD/HC)	Age/Gender matched	Diagnosis	Sample Source	Assay type	Sources of healthy volunteers	BMI (ASD/HC)
Adams JB et al.2006	VB6	USA	11/11	73/90.9	7.2/7.8	Y/N	NA	plasma	NA	Local community	NA
Adams JB et al.2011	VA, VB6, VB9, VB12, VC, VD, VE, Na, Mg, Ca, Fe, Se, Met, Ferritin	USA	55/44	89/89	10/11	Y/Y	NA	plasma, serum	LC-MS, MS, HPLC	Local community	NA
Adams M et al.2007	VB9, VB12, Hcy	USA	17/16	NA	NA	Y/N	DSM-IV, ADOS	serum	CLIA, HPLC	NA	NA
Afrazeh et al. 2015	SOD	Iran	27/18	77.8/55.6	NA	Y/Y	CARS, DSM-IV	serum	ELISA	NA	18.23/20.96
Al-Farsi et al.2013	VB9, VB12, Hcy, Met	Oman	40/40	50/50	4.8/4.8	Y/Y	CARS, DSM-IV-TR	serum	HPLC	Hospital	NA
Al-Gadani et al. 2009	VE, VC, GPx, CAT, SOD, GSH	Saudi Arabia	30/30	73.33/66.67	NA	Y/N	DSM-IV	plasma	Commercially available kits	NA	NA
Ali et al.2011	Hcy, VB9, VB12	Oman	40/40	NA	NA	Y/Y	DSM-IV-TR, CARS	serum	EIA	Hospital	NA
Altun et al. 2018	VD, Hcy, VB6, VB9, VB12, Ca	Turkey	60/45	86.7/80	5.8/6.7	Y/Y	CARS, DSM-IV-TR	serum	ELISA	NA	18.6/19.6

Study/Year	Markers Measured	Country	Samples (ASD/HC)	Gender (%Male) (ASD/HC)	Mean Age (ASD/HC)	Age/Gender matched	Diagnosis	Sample Source	Assay type	Sources of healthy volunteers	BMI (ASD/HC)
Al-Yafee et al. 2011	GSSG, tGSH, GST, GSH/GSSG	Saudi Arabia	20/20	100/100	NA	Y/Y	ADI-R, ADOS, 3DI	plasma	Commercially available kits	Hospital	NA
Arastoo et al.2018	VD	Iran	31/31	83.9/90.3	9.17/9.31	Y/Y	DSM-IV, ADI-R	serum	ELISA	Schools	NA
Bala KA et al.2016	VB9, VB12, VD, Ferritin	Turkey	16/27	62.5/48.1	7.88/9.80	Y/Y	DSM-IV, DSM-V, CARS	serum	CLIA	Hospital	NA
Bener et al.2014	VD, Ca, Mg, Ferritin	UK	254/254	64.9/56.7	NA	Y/N	ADOS, ADI-R	serum	Commercially available kits	Primary Health Care Centers	NA
Bičíková et al. 2019	VD	Czech	45/40	100/100	NA	Y/Y	NA	serum	ECLIA method	NA	NA
Bugajska et al.2017	Met	Poland	27/13	100/100	4.37/5.00	Y/Y	NA	plasma	HPLC-UV/VIS	NA	NA
Cai et al.2016	Hcy	China	51/51	82.4/82.4	3.69/3.69	Y/Y	DSM-IV, CARS	plasma	LC-MS	Kindergarten	NA
Chauhan et al.2004	Cp, Tf, MDA	USA	19/19	NA	4.4/6.0	Y/Y	ADI-R, ADOS-G	serum, plasma	Commercially available kits	Unaffected Siblings	NA
Chen et al. 2016	Hcy, VB12, VD	China	68/68	78/78	3.85/3.85	Y/Y	DSM-V, CARS	serum	Commercially available kits	Kindergarten	26.4/26.2

Study/Year	Markers Measured	Country	Samples (ASD/HC)	Gender (%Male) (ASD/HC)	Mean Age (ASD/HC)	Age/Gender matched	Diagnosis	Sample Source	Assay type	Sources of healthy volunteers	BMI (ASD/HC)
Cheng et al. 2020	VA	China	323/180	84.8/78.9	4.72/4.74	Y/Y	DSM-V, CARS, SRS, ABC	serum	spectrophotometry	Kindergartens and primary school	15.96/16.25
Cortelazzo et al.2016	TG, TC	Italy	30/30	80/66.67	12.0/11.7	Y/Y	DSM-V, ADOS, ABC	plasma	NA	NA	NA
Coşkun et al.2016	VD	Turkey	85/82	84.7/65.8	3.617/3.925	Y/N	DSM-V	serum	ELISA	Local community	NA
D'Eufemia et al.1995	Met	Italy	40/46	67.5/58.6	12.33/11.17	Y/N	DSMIII-R	serum	HPLC	NA	NA
El-Ansary et al. 2010	Ca, K, Na, Mg, MDA	Saudi Arabia	30/30	73.33/66.67	NA	Y/N	DSM-IV	plasma	Commercially available kits	NA	NA
El-Ansary AK et al.2011	Ca, Mg,	Saudi Arabia	25/16	100/100	NA	Y/Y	ADI-R, ADOS, 3DI	plasma	Commercially available kits	Hospital	NA
El-Ansary et al. 2016	GST	Saudi Arabia	20/20	100/100	7/7	Y/Y	ADI-R, ADOS, 3DI	plasma	HPLC, ELISA	Hospital	NA
El-Ansary et al. 2017	VE, VC, GSH, CAT, GPx, SOD	Saudi Arabia	30/30	73.33/66.67	NA	Y/N	ADI-R, ADOS, 3DI	plasma	ELISA	Hospital	NA
El-Ansary et al.2018	VD	Saudi Arabia	28/27	100/100	7.0/7.2	Y/Y	DSM-IV-TR, CARS, SRS	plasma	HPLC, ELISA	Unaffected siblings	NA

**Study/Year**	**Markers Measured**	**Country**	**Samples (ASD/HC)**	**Gender (%Male) (ASD/HC)**	**Mean Age (ASD/HC)**	**Age/Gender matched**	**Diagnosis**	**Sample Source**	**Assay type**	**Sources of healthy volunteers**	**BMI (ASD/HC)**
Essa et al. 2012	MDA, NO	Oman	19/19	78.95/52.63	NA	Y/N	DSM-IV, CARS	plasma	Commercially available kits	Local community	NA
Eto et al.1992	VB9	USA	16/11	63/33	12.31/10.44	N/N	DSM-III R	plasma	HPLC	Local community	NA
Fatemi et al.2002	Cp	USA	28/8	78.5/50	7.4/38.3	N/N	DSM-IV, ADI	serum	NA	Local community	NA
Feng J et al.2016	VD	China	215/285	80.47/78.95	4.76/ 5.12	Y/Y	ABC, CARS	serum	HPLC	Children activity centers	NA
Garipardic et al.2017	Ferritin, VB9, VB12, VD	Turkey	18/25	61.1/48	8.11/9.90	Y/N	DSM-V, DSM-IV-TR, CARS	serum	CLIA	Local community	NA
Geier et al.2009	Cys, GSH, GSSG	USA	38/120	89/	6.0/	Y/N	CARS	plasma	HPLC, LC	Local community	NA
Ghodsi et al. 2019	MDA	Iran	36/18	75/ 77.8	8.64/7.5	Y/Y	DSM-V	plasma	ELISA	NA	NA
Gong et al.2013	VD, Ca, Mg	China	48/48	83.33/83.33	3.67/3.67	Y/Y	DSM-IV, CARS	serum	NA	Kindergarten	15.8/ 16.3
Grayaa et al. 2018	TC, TG	Italy	36/38	63.89/65.79	4.62/4.61	Y/N	DSM-V, CARS	plasma	Commercially available kits	NA	17.21/19.10

Abbreviations: ASD, Autistic Spectrum Disorder; HC, Healthy Control; Y/N, yes/no; BMI, Body Mass Index; VA ,Vitamin A; VB6, Vitamin B6; VB9, Vitamin B9;

Study/Year	Markers Measured	Country	Samples (ASD/HC)	Gender (%Male) (ASD/HC)	Mean Age (ASD/HC)	Age/Gender matched	Diagnosis	Sample Source	Assay type	Sources of healthy volunteers	BMI (ASD/HC)
Gunes et al.2017	Ferritin, Fe	Turkey	100/100	NA	8.36/ 11.01	Y/Y	DSM-V, CARS	serum	NA	Hospital	NA
Guo et al. 2018	VA, VB9, VB12, VD, Ferritin, Ca, Mg, Fe, Zn, Cu	China	274/97	86.13/ 51.54	4.06/ 4.24	Y/N	DSM-V, ABC, SRS, GDS	serum	HPLC	Local community	NA
Guo et al. 2019	VA, VD	China	332/197	86.14/81.72	4.87/4.75	Y/N	DSM-V, ABC, CARS, [illegible]	serum	HPLC	NA	NA
Han et al.2015	Hcy, Cys, tGSH, GSH, GSSG	China	50/50	78/78	7.64/8.38	Y/N	CARS, ABC	serum	Commercially available kits, HPLC	Schools	NA
Hassan et al.2019	TC	Egypt	73/73	100/100	NA	Y/Y	CARS	serum	ELISA, Commercially available kits	Hospital	NA
Hodgson et al. 2014	GSH, Cys, Hcy, SAH, SAM/SAH	USA	27/27	81.5/74.1	5.3/5.5	Y/Y	DSM-IV-TR, CARS	serum	HPLC	Hospital	NA
James et al. 2004	SAM, SAH, SAM/SAH, Hcy, CTH, Cys, GSSG, tGSH, tGSH/GSSG	USA	20/33	70/75.76	6.4/7.4	Y/Y	DSM-IV	plasma	HPLC	NA	NA
James et al.2006	Met, SAM, SAH, SAM/SAH, Hcy, Cys, tGSH, GSSG, CTH, GSH/GSSG, tGSH/GSSG	USA	80/73	89/	7.3/10.8	N/N	DSM-IV, ADOS, CARS	plasma	HPLC	NA	NA
James et al. 2009	Met, SAM, SAH, SAM/SAH, Hcy, Cys,tGSH, GSH, GSSG, GSH/GSSG, tGSH/GSSG	USA	40/42	82/	4.8/4.5	N/N	DSM-IV, CARS	plasma	HPLC	NA	NA

VB12, Vitamin B12; VC, Vitamin C; VD, Vitamin D; VE, Vitamin E; GSH, reduced glutathione; GSSG, oxidized glutathione; tGSH, total glutathione; GPx,

Study/Year	Markers Measured	Country	Sample (ASD/HC)	Gender (%Male) (ASD/HC)	Mean Age (ASD/HC)	Age/Gender matched	Diagnosis	Sample Source	Assay type	Sources of healthy volunteers	BMI (ASD/HC)
Kim et al.2010	TC, TG	South Korea	29/29	100/100	10.1/10.9	Y/Y	DSM-III-R, DSM-IV	plasma	NA	School	20.2/18.6
Kondolot et al.2016	MDA, GPx, SOD, GSH, CAT	Turkey	51/50	78/80	5.8/5.6	Y/Y	DSM-V, DSM IV-TR, CARS	plasma	Commercially available kits, HPLC	Hospital	NA
Li SO et al.2014	Zn, Cu	China	60/60	80/80	3.78/3.78	Y/Y	DSM-IV, CARS	serum	NA	Kindergarten	NA
Main et al. 2014	VB12, Hcy	Australia	35/25	94.3/ 92	7.57/8.56	Y/N	DSM-IVTR	serum	Commercially available kits	Local community	NA
Meguid et al.2010	VD, Ca	Egypt	70/42	NA	5.3/6.1	Y/N	DSM IV, ADI-R	serum	RIA	Hospital	NA
Meguid et al.2011	SOD, GPx, MDA	Egypt	20/25	90/92	4.7/6.0	N/N	DSM-IV-TR, CARS	plasma	Commercially available kits	NA	NA
Melnyk et al.2012	Met, SAM, SAH, SAM/SAH, Hcy, Cys, GSH, GSSG, GSH/GSSG, VB9, VB12	USA	40/54	NA	5.8/6.3	Y/N	DSM-IV, ADOS, CARS	plasma	HPLC-UV	Local community	NA
Mostafa et al. 2010	GPx	Egypt	44/44	30/44	NA	Y/Y	DSM-IV	plasma	Commercially available kits	Hospital	NA
Mostafa et al.2012	VD	Saudi Arabia	50/30	78/80	8.24/8.63	Y/Y	DSM, CARS	serum	ELISA	Unaffected siblings	NA

Glutathione peroxidase; GST, Glutathione-S-transferases; SOD, Superoxide dismutase; MDA, Malondialdehyde; CAT, Catalase; 8-OHdG, 8-hydroxy--2deoxyguanosine; Cys, cysteine; CTH, cystathionine; Hcy, homocysteine; Met, methionine; SAM, S-adenosyl methionine; SAH, S-adenosyl homocysteine; TC,

Study/Year	Markers Measured	Country	Samples (ASD/HC)	Gender (%Male) (ASD/HC)	Mean Age (ASD/HC)	Age/Gender matched	Diagnosis	Sample Source	Assay type	Sources of healthy volunteers	BMI (ASD/HC)
Ning et al. 2018	Hcy	China	102/102	78.43/78.43	4.5/4.5	Y/Y	DSM-V, CARS	serum	ELISA	Kindergarten	16.6/17.4
Oshodi et al. 2017	GSH, SOD, GST, MDA	Nigeria	42/23	71.4/60.9	8.4/	Y/N	DSM-V	plasma	NA	Hospital	NA
Parellada et al.2012	SOD, GSH, CAT, Hcy, VB12, VA, VE, GPx, MDA, Tf, Cp, Cu, Zn, Fe	Spain	35/34	94.2/91.1	12.89 /12.79	Y/Y	DSM-IV, ADOS	plasma	Commercially available kits	Schools	NA
Pasca et al.2006	Hcy, GPx	Romania	12/9	75/67	8.29/8.33	Y/N	DSM-IV	plasma	HPLC	NA	NA
Paşca et al.2009	Met, Hcy, Cys, CTH, VB9, VB12	Romania	39/43	79.4/67.4	7.44/8.31	N/N	DSM-IVR	plasma, serum	GS-MS, Commercially available kits	NA	NA
Ramaekers et al.2013	VB9	Belgium	75/30	81.3/63.3	6.8/7.6	Y/N	NA	Plasma	NA	NA	NA
Rose et al. 2012	GSH, GSSG, GSH/GSSG	USA	38/41	84/49	5.42/6.16	N/N	DSM-IV	plasma	HPLC	Unaffected siblings	NA
Russo et al. 2011	Cu, Zn	USA	152/18	88.15/NA	11.52/NA	Y/Y	DSM-IV, ADI-R	plasma	MS	Health Research Institute/Pfeiffer Treatment Center	NA
Saad et al. 2016	VD	Egypt	122/100	75/75	5.09/4.88	Y/Y	DSM-IV-TR, CARS, ABC	serum	ELISA	Hospitals and unaffected siblings	NA

Total Cholesterol; TG, Triglycerides; Tf, transferrin; Cp, ceruloplasmin; NO, Nitric Oxide; Cu, copper; Zn, zinc; Ca, calcium; Fe, iron; Se, selenium; Mg,

Study/Year	Markers Measured	Country	Samples (ASD/HC)	Gender (%Male) (ASD/HC)	Mean Age (ASD/HC)	Age/Gender matched	Diagnosis	Sample Source	Assay type	Sources of healthy volunteers	BMI (ASD/HC)
Saad et al.2017	VD	Egypt	32/30	68.75/66.7	2.9/3.1	Y/Y	DSM-V, CARS	serum	ELISA	NA	NA
Saha et al.2019	VB9, VB6	India	79/30	82.5/	6.016	Y/Y	DSM IV	plasma	ELISA	NA	NA
Shimmura et al.2011	Met, Cys	Japan	23/22	100/100	13.5/12.2	Y/Y	ADI-R	plasma	HPLC	NA	18.6/17.8
Skalny et al.2017(a)	Cu, Zn, Ca, Mg, Se, Fe	Russia	48/48	NA	6.6/6.5	Y/Y	ICD-10, CARS	serum	Commercially available kits	NA	NA
Skalny et al.2017(b)	Cu, Fe, Se, Zn	Russia	70/70	57.1/57.1	6.4/6.3	Y/Y	ICD-10	serum	Spectrometry	NA	NA
Söğüt et al.2003	SOD, GPx, MDA	Turkey	27/30	59.2/53.3	4.7/5.1	Y/Y	DSM-IV, CARS	plasma	NA	Kindergarten and school	NA
Suh et al.2008	Met, Cys, CTH, SAH, SAM, Hcy, GSH	USA	31/11	87/82	4.2/6.9	N/N	DSM IV, ADI-R	plasma	HPLC, Commercially available kits	Pfeiffer treatment center	NA
Sun et al. 2016	VB9, VB12, Hcy, tGSH, GSSG, tGSH/GSSG	China	29/29	NA	NA	Y/Y	DSM-IV, CARS, ABC	plasma	HPLC	Community Health Care Center	NA
Sweeten et al.2004	NO	USA	29/27	86/85	6.1/6.5	Y/Y	DSM-IV, ADI-R	plasma	Commercially available kits	Local community	NA

Study/Year	Markers Measured	Country	Samples (ASD/HC)	Gender (%Male) (ASD/HC)	Mean Age (ASD/HC)	Age/Gender matched	Diagnosis	Sample Source	Assay type	Sources of healthy volunteers	BMI (ASD/HC)
Sweetman et al.2019	Zn, VA	Ireland	74/72	88/56	9.99/6.43	N/N	DSM-IV, ADOS	serum	MS	NA	NA
Tirouvanziam et al.2011	Met	USA	27/20	77.8/45	7/7.33	Y/N	ADI-R, ADOS	plasma	LC–MS	Local community	NA
Tostes et al.2012	NO	Brazil	24/24	75/75	7.4/7.2	Y/Y	DSM-IV	plasma	ELISA	School	NA
Tu et al.2013	Hcy, VB9, VB12	China	30/30	83.33/83.33	3.55 /3.55	Y/Y	DSM-IV	serum	Chemiluminescence immunoassay	NA	NA
Vergani et al.2011	Ca, Zn, Cu, Fe	Italy	28/32	75/63	NA	N/N	DSM-IV	plasma	ICP-AES	NA	NA
Wang et al.2016	SOD, Hcy	China	98/98	79.6/79.6	3.85/3.85	Y/Y	DSM-V, CARS	serum	NA	Kindergarten	16.22 /16.93
Yektas et al.2019	VB12, VB9, Hcy	Turkey	35/35	80/100	NA	N/N	DSM-IV, CARS	serum	Spectrometry	NA	NA
Yenkoyan et al. 2018	SOD, CAT, MDA	Armenia	10/10	40/40	NA	Y/Y	ADI-R, ADOS	plasma	Spectrometry	Unaffected siblings	NA
Yorbik et al.2002	SOD, GPx	Turkey	45/41	87/85	6.4/6.7	Y/Y	DSM IV	Plasma	Spectrometry	School	NA

Study/Year	Markers Measured	Country	Samples (ASD/HC)	Gender (%Male) (ASD/HC)	Mean Age (ASD/HC)	Age/Gender matched	Diagnosis	Sample Source	Assay type	Sources of healthy volunteers	BMI (ASD/HC)
Yui et al. 2016(a)	SOD	Japan	20/12	65/66.67	11.1/14.3	Y/Y	DSM-V, ADI-R	plasma	Commercially available kits	Local community	NA
Yui et al.2016(b)	Cp, Tf, SOD	Japan	28/21	71.4/71.4	13.5/13.9	Y/Y	DSM-IV-TR, ADI-R	plasma	Commercially available kits	Local community	NA
Yui et al.2016(c)	SOD, Cp, Tf	Japan	30/20	66.67/70	13.6/13.2	Y/Y	DSM-IV-TR, ADI-R	plasma	Commercially available kits	Local community	NA
Yui et al. 2017	SOD	Japan	20/11	65/63.63	10.7/14.7	Y/Y	DSM-V, ADI-R, ADOS	plasma	Commercially available kits	NA	NA
Zaki et al.2017	Met, Cys,	Egypt	42/26	81/81	NA	Y/Y	DSM-IV, ADI-R	plasma	HPLC	NA	NA
Zhang et al.2015	Hcy	China	80/100	78.75/79	3.82/3.79	Y/Y	CARS	serum	Commercially available kits	Kindergarten	15.52/16.83
Zhou W et al.2018	VA, Hcy	China	81/81	79/79	3.8/3.8	Y/Y	DSM-V, CARS	serum	ELISA	NA	16.8/17.7

magnesium; DSM-IV-TR, Diagnostic and Statistical Manual of Mental Disorders-4th Edition Text Revision; ADI-R, Autism Diagnostic Interview–Revised; ADOS, Autism Diagnostic Observation Schedule; 3DI, Developmental, dimensional diagnostic interview; ABC, Autism Behavior Checklist; CARS, Childhood Autism Rating Scale; HPLC, High Performance Liquid Chromatography; ELISA, enzyme linked immunosorbent assay; LC-MS, liquid chromatography-tandem mass spectroscopy; CLIA, Chemiluminescence method; EIA, enzyme immunoassay; HPLC-UV/Vis, High Performance Liquid Chromatography-Visible Spectrophotometer; RIA, Radioimmunoassay; GS-MS, gas chromatography/mass spectrometry; ICP-AES, Inductively Coupled Plasma Atomic Emission

Spectrometer.

eTable 2. Newcastle-Ottawa quality assessment scale for included studies.

Between-Group Studies	Selection	Comparability	Exposure	Total
Adams JB et al.2006	***	*	*	5
Adams JB et al.2011	***	**	**	7
Adams M et al.2007	**	*	*	4
Afrazeh et al. 2015	****	**	**	8
Al-Farsi et al.2013	****	**	**	8
Al-Gadani et al. 2009	*	**	**	5
Ali et al.2011	***	**	**	7
Altun et al. 2018	**	**	**	6
Al-Yafee et al. 2011	***	**	**	7
Arastoo et al.2018	***	**	**	7
Bala KA et al.2016	***	**	*	6
Bener et al.2014	**	**	*	5
Bičíková et al. 2019	*	**	*	4
Bugajska et al.2017	**	**	*	5
Cai et al.2016	***	**	*	6
Chauhan et al.2004	**	**	*	5
Chen et al. 2016	****	**	**	8
Cheng et al. 2020	****	**	**	8
Cortelazzo et al.2016	***	**	**	7
Coşkun et al.2016	***	**	*	6
D'Eufemia et al.1995	**	**	*	5
El-Ansary AK et al.2011	***	**	**	7
El-Ansary et al. 2010	*	**	*	4
El-Ansary et al. 2016	***	**	*	6
El-Ansary et al. 2017	***	**	**	7
El-Ansary et al.2018	***	**	**	7
Essa et al. 2012	**	**	**	6
Eto et al.1992	****	**	**	8
Fatemi et al.2002	*	**	**	5
Feng J et al.2016	****	**	**	8
Garipardic et al.2017	***	*	**	6
Geier et al.2009	**	*	**	5
Ghodsi et al. 2019	*	*	**	4
Gong et al.2013	****	**	**	8
Grayaa et al. 2018	**	**	**	6
Gunes et al.2017	***	*	**	6
Guo et al. 2018	****	*	**	7
Guo et al. 2019	***	*	*	5

Between-Group Studies	Selection	Comparability	Exposure	Total
Han et al.2015	****	**	**	8
Hassan et al.2019	**	**	**	6
Hodgson et al. 2014	***	**	**	7
James et al. 2004	***	**	**	7
James et al.2006	**	**	*	5
James et al. 2009	**	*	*	4
Kim et al.2010	***	**	*	6
Kondolot et al.2016	***	**	**	7
Li SO et al.2014	**	**	**	6
Main et al. 2014	*	*	*	3
Meguid et al.2010	****	*	**	7
Meguid et al.2011	***	**	**	7
Melnyk et al.2012	***	*	**	6
Mostafa et al. 2010	**	**	**	6
Mostafa et al.2012	**	**	*	5
Ning et al. 2018	****	**	*	7
Oshodi et al. 2017	***	**	*	6
Parellada et al.2012	***	**	*	6
Pasca et al.2006	*	**	**	5
Paşca et al.2009	***	**	**	7
Ramaekers et al.2013	*	**	*	4
Rose et al. 2012	**	**	*	5
Russo et al. 2011	***	**	*	6
Saad et al. 2016	***	**	*	6
Saad et al.2017	**	**	**	6
Saha et al.2019	**	*	**	5
Shimmura et al.2011	**	**	*	5
Skalny et al.2017(a)	***	**	**	7
Skalny et al.2017(b)	*	**	**	5
Söğüt et al.2003	**	**	**	6
Suh et al.2008	**	**	*	5
Sun et al. 2016	***	**	**	7
Sweeten et al.2004	***	**	*	6
Sweetman et al.2019	***	**	*	6
Tirouvanziam et al.2011	***	**	*	6
Tostes et al.2012	*	**	*	4
Tu et al.2013	***	**	*	6
Vergani et al.2011	*	**	*	4
Wang et al.2016	***	**	**	7
Yektas et al.2019	**	*	**	5

Between-Group Studies	Selection	Comparability	Exposure	Total
Yenkoyan et al. 2018	**	**	*	5
Yorbik et al.2002	**	**	*	5
Yui et al. 2016(a)	***	**	**	7
Yui et al.2016(b)	****	**	**	8
Yui et al.2016(c)	***	**	*	6
Yui et al. 2017	**	**	*	5
Zaki et al.2017	***	**	*	6
Zhang et al.2015	**	**	*	5
Zhou W et al.2018	***	**	**	7

eTable 3. Sub-group analysis stratified by source of healthy volunteer for homocysteine, methionine, vitamin B9, vitamin B12, vitamin D and GSH.

Marker	Subgroup	Main Effect			Heterogeneity			
		Hedges *g* (95% CI)	*Z* Score	*P* Value	*Q* Statistic	*df*	*P* Value	I^2 Statistic
Homocysteine	Clinical center	0.874(0.618 to 1.131)	6.684	<.001	158.361	4	<.001	97.474
	Kindergarten/Primary School	0.558(0.428 to 0.688)	8.404	<.001	74.798	6	<.001	91.978
Methionine	Local community	-0.319(-0.576 to -0.062)	-2.429	0.015	9.480	2	0.009	78.903
Vitamin B9	Clinical center	-0.897(-1.188 to -0.606)	-6.038	<.001	126.912	3	<.001	97.636
	Local community	-0.232(-0.400 to -0.064)	-2.705	0.007	7.677	4	0.104	47.896
Vitamin B12	Clinical center	-0.520(-0.768 to -0.272)	-4.106	<.001	13.291	3	0.004	77.429
	Local community	0.052(-0.111 to 0.216)	0.628	0.530	19.837	4	0.001	79.836
Vitamin D	Clinical center	-0.382(-0.499 to -0.265)	-6.389	<.001	18.428	3	<.001	83.720
	Kindergarten/Primary School	-0.677(-0.911 to -0.444)	-5.682	<.001	1.635	2	0.441	0.000
	Local community	-0.060(-0.223 to 0.103)	-0.721	0.471	44.361	3	<.001	93.237
GSH	Clinical center	-0.292(-0.542 to -0.043)	-2.294	0.022	6.028	3	0.110	50.235
	Local community	-1.141(-1380 to -0.901)	-9.316	<.001	12.806	2	0.002	84.382

Abbreviations: df, degrees of freedom; ASD, Autism spectrum disorder; GSH, Reduced glutathione.

eFigure 1

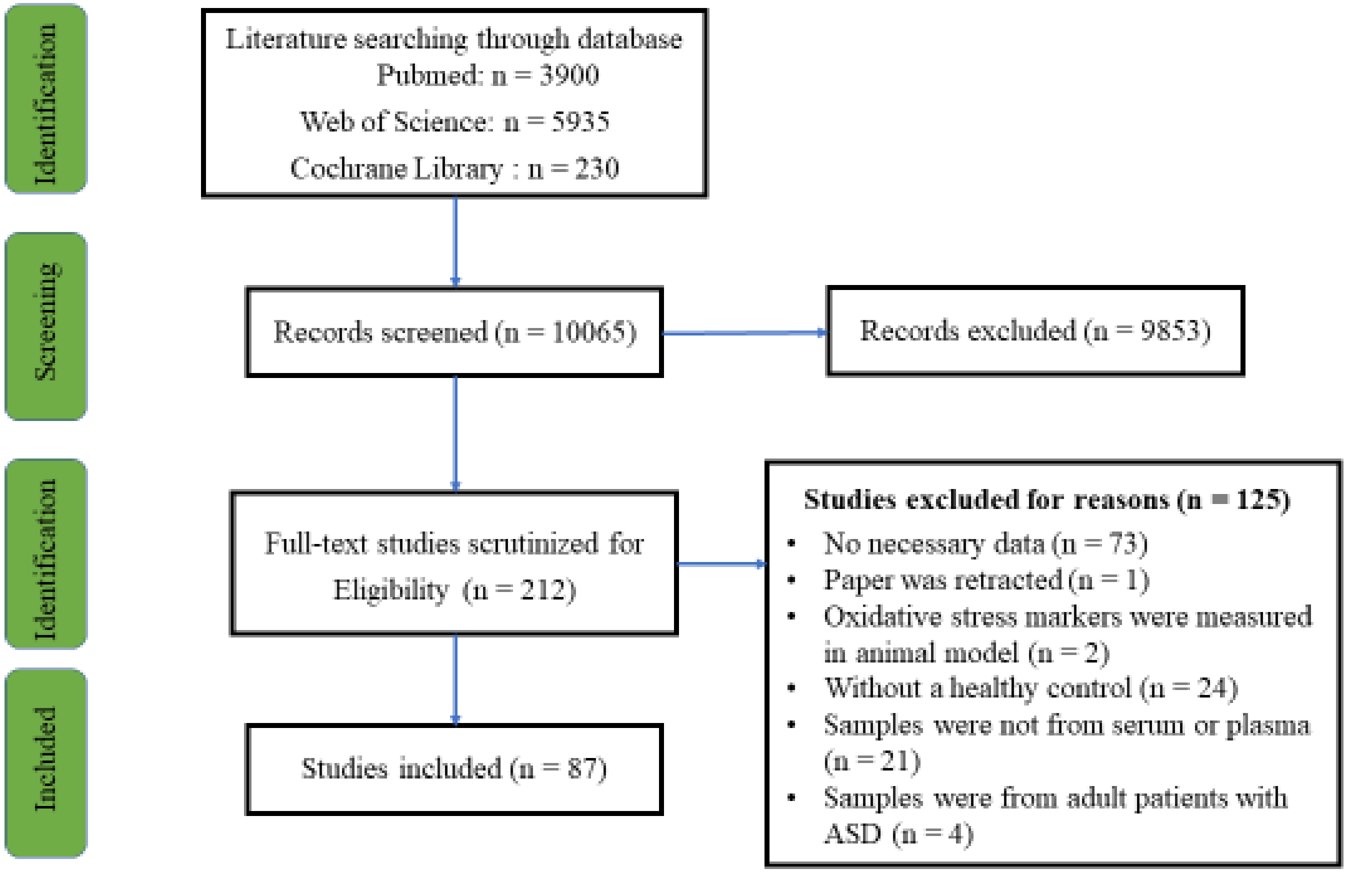

Identification
Screening
Identification
Included
Literature searching through database
Pubmed: n = 3900
Web of Science: n = 5935
Cochrane Library : n = 230
Records screened (n = 10065)
Records excluded (n = 9853)
Full-text studies scrutinized for Eligibility (n = 212)
Studies excluded for reasons (n = 125)
• No necessary data (n = 73)
• Paper was retracted (n = 1)
• Oxidative stress markers were measured in animal model (n = 2)
• Without a healthy control (n = 24)
• Samples were not from serum or plasma (n = 21)
• Samples were from adult patients with ASD (n = 4)
Studies included (n = 87)

eFigure 2

A

Homocysteine

Group by Source	Study name	Hedges's g	Lower limit	Upper limit	p-Value
plasma	Cai et al.2016	2.616	2.090	3.143	0.000
plasma	James et al. 2004	-0.494	-1.049	0.061	0.081
plasma	James et al.2006	-0.239	-0.556	0.078	0.139
plasma	James et al. 2009	-0.130	-0.560	0.299	0.552
plasma	Melnyk et al.2012	0.136	-0.270	0.542	0.510
plasma	Parellada et al.2012	-0.128	-0.651	0.394	0.631
plasma	Pasca et al.2006	1.023	0.137	1.908	0.024
plasma	Pasca et al.2009	-0.331	-0.763	0.101	0.133
plasma	Suh et al.2008	-0.492	-1.175	0.191	0.158
plasma	Sun et al. 2016	0.546	0.029	1.064	0.038
plasma		0.238	-0.305	0.780	0.390
serum	Adams et al.2007	0.240	-0.429	0.909	0.482
serum	Al-Farsi et al.2013	0.657	0.211	1.103	0.004
serum	Ali et al.2011	3.745	3.021	4.470	0.000
serum	Altun et al. 2018	7.191	6.145	8.236	0.000
serum	Chen et al. 2016	0.082	-0.252	0.417	0.629
serum	Han et al.2015	0.447	0.053	0.841	0.026
serum	Hodgson et al. 2014	0.717	0.174	1.260	0.010
serum	Main et al. 2014	0.394	-0.118	0.905	0.131
serum	Ning et al. 2018	0.696	0.415	0.978	0.000
serum	Tu et al.2013	1.399	0.840	1.958	0.000
serum	Wang et al.2016	0.475	0.193	0.758	0.001
serum	Yektas et al.2019	0.813	0.331	1.296	0.001
serum	Zhang et al.2015	0.500	0.203	0.797	0.001
serum	Zhou W et al.2018	1.073	0.745	1.401	0.000
serum		1.206	0.719	1.693	0.000

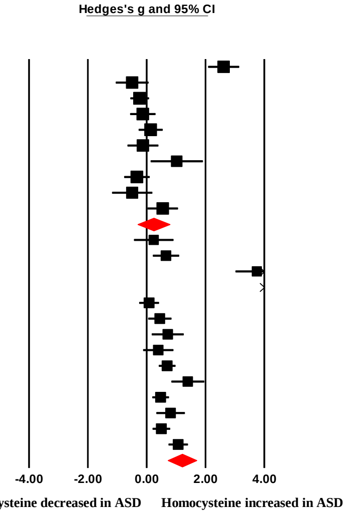

Homocysteine

Group by Age matching	Study name	Hedges's g	Lower limit	Upper limit	p-Value
N	James et al. 2004	-0.494	-1.049	0.061	0.081
N	James et al.2006	-0.239	-0.556	0.078	0.139
N	Pasca et al.2009	-0.331	-0.763	0.101	0.133
N	Suh et al.2008	-0.492	-1.175	0.191	0.158
N	Yektas et al.2019	0.813	0.331	1.296	0.001
N		-0.135	-0.591	0.320	0.560
Y	Adams et al.2007	0.240	-0.429	0.909	0.482
Y	Al-Farsi et al.2013	0.657	0.211	1.103	0.004
Y	Ali et al.2011	3.745	3.021	4.470	0.000
Y	Altun et al. 2018	7.191	6.145	8.236	0.000
Y	Cai et al.2016	2.616	2.090	3.143	0.000
Y	Chen et al. 2016	0.082	-0.252	0.417	0.629
Y	Han et al.2015	0.447	0.053	0.841	0.026
Y	Hodgson et al. 2014	0.717	0.174	1.260	0.010
Y	James et al. 2009	-0.130	-0.560	0.299	0.552
Y	Main et al. 2014	0.394	-0.118	0.905	0.131
Y	Melnyk et al.2012	0.136	-0.270	0.542	0.510
Y	Ning et al. 2018	0.696	0.415	0.978	0.000
Y	Parellada et al.2012	-0.128	-0.651	0.394	0.631
Y	Pasca et al.2006	1.023	0.137	1.908	0.024
Y	Sun et al. 2016	0.546	0.029	1.064	0.038
Y	Tu et al.2013	1.399	0.840	1.958	0.000
Y	Wang et al.2016	0.475	0.193	0.758	0.001
Y	Zhang et al.2015	0.500	0.203	0.797	0.001
Y	Zhou W et al.2018	1.073	0.745	1.401	0.000
Y		1.060	0.631	1.490	0.000

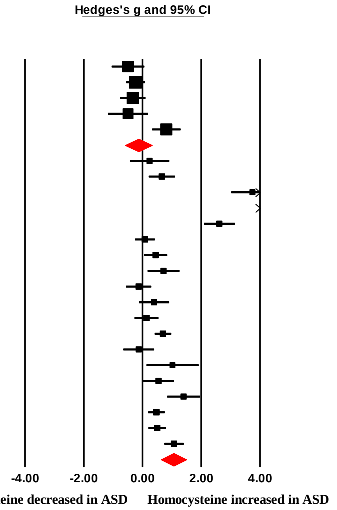

Homocysteine

Group by Gender matching	Study name	Hedges's g	Lower limit	Upper limit	p-Value
N	Adams et al.2007	0.240	-0.429	0.909	0.482
N	James et al. 2004	-0.494	-1.049	0.061	0.081
N	James et al.2006	-0.239	-0.556	0.078	0.139
N	James et al. 2009	-0.130	-0.560	0.299	0.552
N	Main et al. 2014	0.394	-0.118	0.905	0.131
N	Melnyk et al.2012	0.136	-0.270	0.542	0.510
N	Pasca et al.2006	1.023	0.137	1.908	0.024
N	Pasca et al.2009	-0.331	-0.763	0.101	0.133
N	Suh et al.2008	-0.492	-1.175	0.191	0.158
N	Yektas et al.2019	0.813	0.331	1.296	0.001
N		0.055	-0.229	0.338	0.706
Y	Al-Farsi et al.2013	0.657	0.211	1.103	0.004
Y	Ali et al.2011	3.745	3.021	4.470	0.000
Y	Altun et al. 2018	7.191	6.145	8.236	0.000
Y	Cai et al.2016	2.616	2.090	3.143	0.000
Y	Chen et al. 2016	0.082	-0.252	0.417	0.629
Y	Han et al.2015	0.447	0.053	0.841	0.026
Y	Hodgson et al. 2014	0.717	0.174	1.260	0.010
Y	Ning et al. 2018	0.696	0.415	0.978	0.000
Y	Parellada et al.2012	-0.128	-0.651	0.394	0.631
Y	Sun et al. 2016	0.546	0.029	1.064	0.038
Y	Tu et al.2013	1.399	0.840	1.958	0.000
Y	Wang et al.2016	0.475	0.193	0.758	0.001
Y	Zhang et al.2015	0.500	0.203	0.797	0.001
Y	Zhou W et al.2018	1.073	0.745	1.401	0.000
Y		1.333	0.799	1.866	0.000

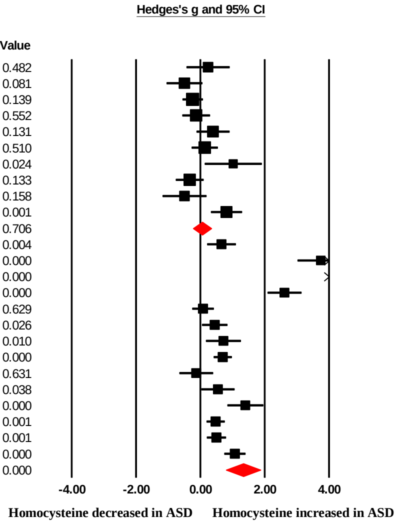

Forrest plot showing pooled results comparing homocysteine levels between ASD patients and HC subjects stratified by source of sampling (plasma heterogeneity: Q = 111.997, P < 0.001, I^2 = 91.964, 10 studies; and serum heterogeneity: Q = 248.685, P < 0.001, I^2 = 94.773, 14 studies), age matching (N heterogeneity: Q = 18.688, P = 0.001, I^2 = 78.596, 5 studies; and Y heterogeneity: Q = 332.029, P < 0.001, I^2 = 94.579, 19 studies) and gender matching (N heterogeneity: Q = 29.061, P < 0.001, I^2 = 69.031, 10 studies; and Y heterogeneity: Q = 303.569, P < 0.001, I^2 = 95.718, 14 studies). The sizes of the squares are proportional to study weights.

eFigure 2

B

Methionine

Group by Source	Study name	Hedges's g	Lower limit	Upper limit	p-Value
plasma	Adams et al.2011	0.150	-0.244	0.544	0.455
plasma	Bugajska et al.2017	-0.203	-0.853	0.447	0.540
plasma	James et al. 2004	-1.614	-2.242	-0.987	0.000
plasma	James et al.2006	-1.257	-1.603	-0.912	0.000
plasma	James et al. 2009	-0.844	-1.291	-0.396	0.000
plasma	Melnyk et al.2012	-0.674	-1.091	-0.258	0.002
plasma	Pasca et al.2009	-0.830	-1.278	-0.383	0.000
plasma	Shimmura et al.2011	0.385	-0.195	0.965	0.193
plasma	Tirouvanziam et al.2011	-0.650	-1.234	-0.067	0.029
plasma	Zaki et al.2017	-0.850	-1.354	-0.345	0.001
plasma		-0.643	-1.009	-0.276	0.001

Statistics for each study

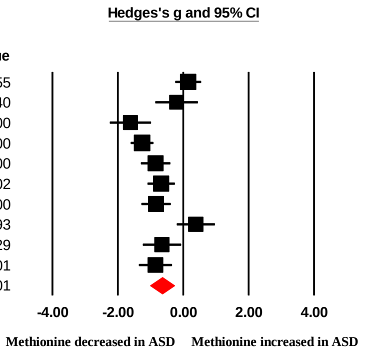

Methionine

Group by Age matching	Study name	Hedges's g	Lower limit	Upper limit	p-Value
N	James et al. 2004	-1.614	-2.242	-0.987	0.000
N	James et al.2006	-1.257	-1.603	-0.912	0.000
N	Pasca et al.2009	-0.830	-1.278	-0.383	0.000
N		-1.197	-1.589	-0.804	0.000
Y	Adams et al.2011	0.150	-0.244	0.544	0.455
Y	Al-Farsi et al.2013	-0.441	-0.880	-0.001	0.049
Y	Bugajska et al.2017	-0.203	-0.853	0.447	0.540
Y	D'Eufemia et al.1995	-0.119	-0.539	0.301	0.579
Y	James et al. 2009	-0.844	-1.291	-0.396	0.000
Y	Melnyk et al.2012	-0.674	-1.091	-0.258	0.002
Y	Shimmura et al.2011	0.385	-0.195	0.965	0.193
Y	Tirouvanziam et al.2011	-0.650	-1.234	-0.067	0.029
Y	Zaki et al.2017	-0.850	-1.354	-0.345	0.001
Y		-0.366	-0.653	-0.079	0.012

Statistics for each study

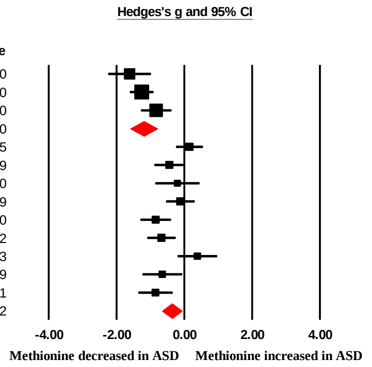

Methionine

Group by Gender matching	Study name	Hedges's g	Lower limit	Upper limit	p-Value
N	D'Eufemia et al.1995	-0.119	-0.539	0.301	0.579
N	James et al. 2004	-1.614	-2.242	-0.987	0.000
N	James et al.2006	-1.257	-1.603	-0.912	0.000
N	James et al. 2009	-0.844	-1.291	-0.396	0.000
N	Melnyk et al.2012	-0.674	-1.091	-0.258	0.002
N	Pasca et al.2009	-0.830	-1.278	-0.383	0.000
N	Tirouvanziam et al.2011	-0.650	-1.234	-0.067	0.029
N		-0.841	-1.183	-0.498	0.000
Y	Adams et al.2011	0.150	-0.244	0.544	0.455
Y	Al-Farsi et al.2013	-0.441	-0.880	-0.001	0.049
Y	Bugajska et al.2017	-0.203	-0.853	0.447	0.540
Y	Shimmura et al.2011	0.385	-0.195	0.965	0.193
Y	Zaki et al.2017	-0.850	-1.354	-0.345	0.001
Y		-0.196	-0.623	0.230	0.367

Statistics for each study

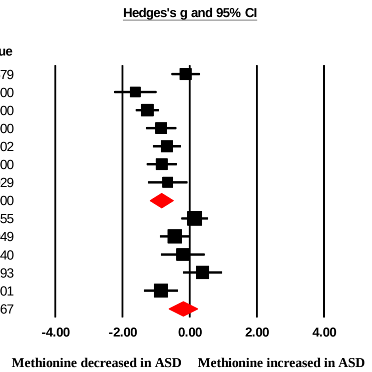

Forrest plot showing pooled results comparing plasma methionine levels (heterogeneity Q = 52.702, P < 0.001, I^2 = 82.923, 10 studies), age matching (N heterogeneity: Q = 4.375, P = 0.112, I^2 = 54.291, 3 studies; and Y heterogeneity: Q = 25.631, P = 0.001, I^2 = 68.788, 9 studies) and gender matching (N heterogeneity: Q = 23.760, P = 0.001, I^2 = 74.747, 7 studies; and Y heterogeneity: Q = 14.472, P = 0.006, I^2 = 72.361, 5 studies) between ASD patients and HC subjects. The sizes of the squares are proportional to study weights.

eFigure 2

C

Cysteine

Group by Source	Study name	Hedges's g	Lower limit	Upper limit	p-Value
		Statistics for each study			
plasma	Geier et al.2009	-0.879	-1.299	-0.459	0.000
plasma	James et al. 2004	-2.360	-3.068	-1.652	0.000
plasma	James et al.2006	-2.289	-2.696	-1.882	0.000
plasma	James et al. 2009	-0.890	-1.340	-0.440	0.000
plasma	Melnyk et al.2012	-0.578	-0.992	-0.165	0.006
plasma	Pasca et al.2009	-0.296	-0.728	0.135	0.178
plasma	Shimmura et al.2011	0.162	-0.414	0.737	0.582
plasma	Suh et al.2008	-0.019	-0.693	0.656	0.957
plasma	Zaki et al.2017	-0.736	-1.235	-0.237	0.004
plasma		-0.875	-1.422	-0.328	0.002

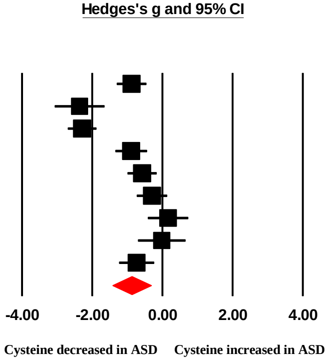

Cysteine

Group by Age matching	Study name	Hedges's g	Lower limit	Upper limit	p-Value
		Statistics for each study			
N	James et al. 2004	-2.360	-3.068	-1.652	0.000
N	James et al.2006	-2.289	-2.696	-1.882	0.000
N	Pasca et al.2009	-0.296	-0.728	0.135	0.178
N	Suh et al.2008	-0.019	-0.693	0.656	0.957
N		-1.241	-2.478	-0.004	0.049
Y	Geier et al.2009	-0.879	-1.299	-0.459	0.000
Y	Han et al.2015	-0.559	-0.955	-0.162	0.006
Y	Hodgson et al. 2014	0.541	0.005	1.076	0.048
Y	James et al. 2009	-0.890	-1.340	-0.440	0.000
Y	Melnyk et al.2012	-0.578	-0.992	-0.165	0.006
Y	Shimmura et al.2011	0.162	-0.414	0.737	0.582
Y	Zaki et al.2017	-0.736	-1.235	-0.237	0.004
Y		-0.443	-0.812	-0.074	0.018

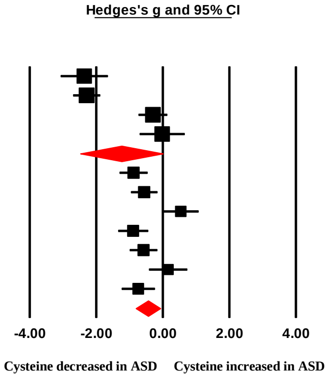

Cysteine

Group by Gender matching	Study name	Hedges's g	Lower limit	Upper limit	p-Value
		Statistics for each study			
N	Geier et al.2009	-0.879	-1.299	-0.459	0.000
N	James et al. 2004	-2.360	-3.068	-1.652	0.000
N	James et al.2006	-2.289	-2.696	-1.882	0.000
N	James et al. 2009	-0.890	-1.340	-0.440	0.000
N	Melnyk et al.2012	-0.578	-0.992	-0.165	0.006
N	Pasca et al.2009	-0.296	-0.728	0.135	0.178
N	Suh et al.2008	-0.019	-0.693	0.656	0.957
N		-1.039	-1.677	-0.402	0.001
Y	Han et al.2015	-0.559	-0.955	-0.162	0.006
Y	Hodgson et al. 2014	0.541	0.005	1.076	0.048
Y	Shimmura et al.2011	0.162	-0.414	0.737	0.582
Y	Zaki et al.2017	-0.736	-1.235	-0.237	0.004
Y		-0.165	-0.743	0.413	0.576

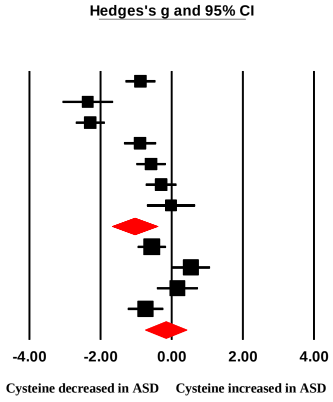

Forrest plot showing pooled results comparing plasma Cysteine levels (heterogeneity Q = 91.02, P < 0.001, I^2 = 91.211, 9 studies), age matching (N heterogeneity: Q = 65.923, P < 0.001, I^2 = 95.449, 4 studies; and Y heterogeneity: Q = 26.673, P < 0.001, , I^2 = 77.505, 7 studies) and gender matching (N heterogeneity: Q = 75.520, P < 0.001, I^2 = 92.055, 7 studies; and Y heterogeneity: Q = 16.306, P = 0.001, I^2 = 81.602, 4 studies) between ASD patients and HC subjects. The sizes of the squares are proportional to study weights.

eFigure 2

D

Vitamin B9

Group by Source	Study name	Hedges's g	Lower limit	Upper limit	p-Value
serum	Adams JB et al.2011	-0.147	-0.541	0.247	0.463
serum	Adams M et al.2007	0.071	-0.595	0.737	0.835
serum	Al-Farsi et al.2013	-0.757	-1.207	-0.308	0.001
serum	Ali et al.2011	-8.352	-9.717	-6.987	0.000
serum	Altun et al. 2018	-3.974	-4.634	-3.314	0.000
serum	Bala KA et al.2016	0.167	-0.441	0.775	0.591
serum	Garipardic et al.2017	0.167	-0.429	0.763	0.582
serum	Guo et al. 2018	-0.352	-0.585	-0.120	0.003
serum	Pasca et al.2009	0.317	-0.115	0.749	0.151
serum	Ramaekers et al.2013	0.427	0.003	0.852	0.048
serum	Tu et al.2013	-4.447	-5.386	-3.507	0.000
serum	Yektas et al.2019	0.813	0.331	1.296	0.001
serum		-1.213	-2.033	-0.393	0.004

Statistics for each study

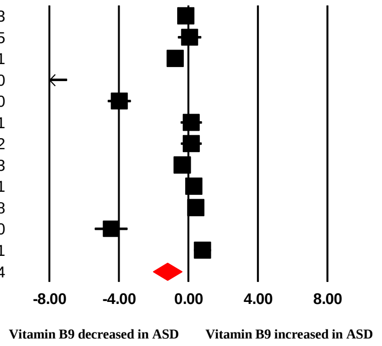

Vitamin B9

Group by Age matching	Study name	Hedges's g	Lower limit	Upper limit	p-Value
N	Eto et al.1992	0.573	-0.187	1.332	0.140
N	Pasca et al.2009	0.317	-0.115	0.749	0.151
N	Yektas et al.2019	0.813	0.331	1.296	0.001
N		0.547	0.227	0.867	0.001
Y	Adams JB et al.2011	-0.147	-0.541	0.247	0.463
Y	Adams M et al.2007	0.071	-0.595	0.737	0.835
Y	Al-Farsi et al.2013	-0.757	-1.207	-0.308	0.001
Y	Ali et al.2011	-8.352	-9.717	-6.987	0.000
Y	Altun et al. 2018	-3.974	-4.634	-3.314	0.000
Y	Bala KA et al.2016	0.167	-0.441	0.775	0.591
Y	Garipardic et al.2017	0.167	-0.429	0.763	0.582
Y	Guo et al. 2018	-0.352	-0.585	-0.120	0.003
Y	Melnyk et al.2012	-0.371	-0.779	0.038	0.076
Y	Ramaekers et al.2013	0.427	0.003	0.852	0.048
Y	Saha et al.2019	0.062	-0.356	0.479	0.772
Y	Sun et al. 2016	-0.777	-1.305	-0.250	0.004
Y	Tu et al.2013	-4.447	-5.386	-3.507	0.000
Y		-1.270	-1.983	-0.556	0.000

Statistics for each study

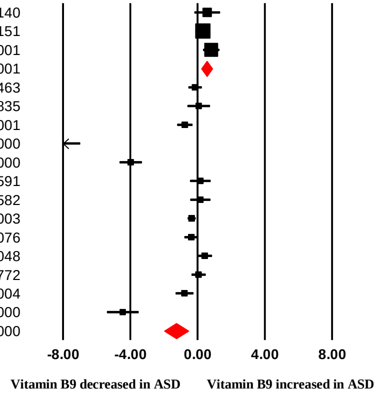

Vitamin B9

Group by Gender matching	Study name	Hedges's g	Lower limit	Upper limit	p-Value
N	Adams M et al.2007	0.071	-0.595	0.737	0.835
N	Eto et al.1992	0.573	-0.187	1.332	0.140
N	Garipardic et al.2017	0.167	-0.429	0.763	0.582
N	Guo et al. 2018	-0.352	-0.585	-0.120	0.003
N	Melnyk et al.2012	-0.371	-0.779	0.038	0.076
N	Pasca et al.2009	0.317	-0.115	0.749	0.151
N	Ramaekers et al.2013	0.427	0.003	0.852	0.048
N	Yektas et al.2019	0.813	0.331	1.296	0.001
N		0.176	-0.163	0.515	0.309
Y	Adams JB et al.2011	-0.147	-0.541	0.247	0.463
Y	Al-Farsi et al.2013	-0.757	-1.207	-0.308	0.001
Y	Ali et al.2011	-8.352	-9.717	-6.987	0.000
Y	Altun et al. 2018	-3.974	-4.634	-3.314	0.000
Y	Bala KA et al.2016	0.167	-0.441	0.775	0.591
Y	Saha et al.2019	0.062	-0.356	0.479	0.772
Y	Sun et al. 2016	-0.777	-1.305	-0.250	0.004
Y	Tu et al.2013	-4.447	-5.386	-3.507	0.000
Y		-2.176	-3.468	-0.885	0.001

Statistics for each study

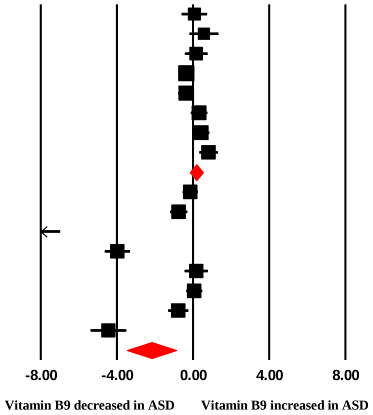

Forrest plot showing pooled results comparing serum vitamin B9 levels (heterogeneity: Q = 376.382, P < 0.001, I^2 = 97.077, 12 studies), age matching (N heterogeneity: Q = 2.263, P = 0.323, I^2 = 11.631, 3 studies; and Y heterogeneity: Q = 346.128, P < 0.001, I^2 = 96.533, 13 studies) and gender matching (N heterogeneity: Q = 31.353, P < 0.001, I^2 = 77.674, 8 studies; and Y heterogeneity: Q = 299.251, P < 0.001, I^2 = 97.661, 8 studies) between ASD patients and HC subjects. The sizes of the squares are proportional to study weights.

eFigure 2

E

Vitamin B12

Group by Source	Study name	Hedges's g	Lower limit	Upper limit	p-Value
plasma	Adams JB et al.2011	0.101	-0.293	0.494	0.616
plasma	Melnyk et al.2012	0.015	-0.391	0.420	0.944
plasma	Parellada et al.2012	-0.126	-0.593	0.341	0.598
plasma	Sun et al. 2016	0.143	-0.365	0.652	0.581
plasma	Yektas et al.2019	-0.813	-1.296	-0.331	0.001
plasma		-0.127	-0.453	0.199	0.445
serum	Adams M et al.2007	0.482	-0.194	1.158	0.162
serum	Al-Farsi et al.2013	-0.757	-1.207	-0.308	0.001
serum	Ali et al.2011	-0.441	-0.880	-0.001	0.049
serum	Altun et al. 2018	-3.538	-4.152	-2.925	0.000
serum	Bala KA et al.2016	-1.324	-1.993	-0.656	0.000
serum	Chen et al. 2016	0.138	-0.197	0.473	0.419
serum	Garipardic et al.2017	-1.382	-2.045	-0.720	0.000
serum	Guo et al. 2018	0.191	-0.040	0.423	0.106
serum	Main et al. 2014	0.207	-0.301	0.715	0.425
serum	Pasca et al.2009	-0.203	-0.634	0.227	0.354
serum	Tu et al.2013	-0.564	-1.074	-0.055	0.030
serum		-0.634	-1.183	-0.086	0.023

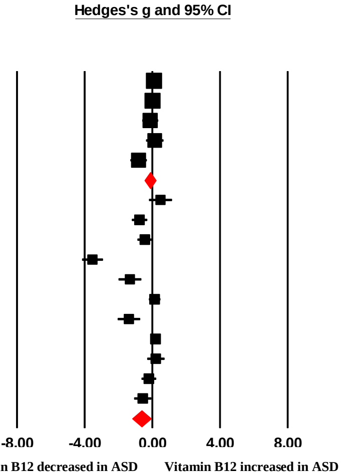

Vitamin B12

Group by Age matching	Study name	Hedges's g	Lower limit	Upper limit	p-Value
Y	Adams JB et al.2011	0.101	-0.293	0.494	0.616
Y	Adams M et al.2007	0.482	-0.194	1.158	0.162
Y	Al-Farsi et al.2013	-0.757	-1.207	-0.308	0.001
Y	Ali et al.2011	-0.441	-0.880	-0.001	0.049
Y	Altun et al. 2018	-3.538	-4.152	-2.925	0.000
Y	Bala KA et al.2016	-1.324	-1.993	-0.656	0.000
Y	Chen et al. 2016	0.138	-0.197	0.473	0.419
Y	Garipardic et al.2017	-1.382	-2.045	-0.720	0.000
Y	Guo et al. 2018	0.191	-0.040	0.423	0.106
Y	Main et al. 2014	0.207	-0.301	0.715	0.425
Y	Melnyk et al.2012	0.015	-0.391	0.420	0.944
Y	Parellada et al.2012	-0.126	-0.593	0.341	0.598
Y	Sun et al. 2016	0.143	-0.365	0.652	0.581
Y	Tu et al.2013	-0.564	-1.074	-0.055	0.030
Y		-0.467	-0.900	-0.035	0.034

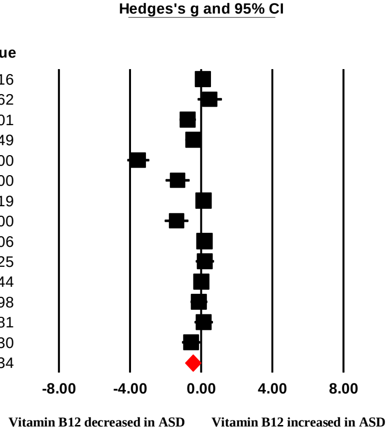

Vitamin B12

Group by Gender matching	Study name	Hedges's g	Lower limit	Upper limit	p-Value
N	Adams M et al.2007	0.482	-0.194	1.158	0.162
N	Garipardic et al.2017	-1.382	-2.045	-0.720	0.000
N	Guo et al. 2018	0.191	-0.040	0.423	0.106
N	Main et al. 2014	0.207	-0.301	0.715	0.425
N	Melnyk et al.2012	0.015	-0.391	0.420	0.944
N	Pasca et al.2009	-0.203	-0.634	0.227	0.354
N	Yektas et al.2019	-0.813	-1.296	-0.331	0.001
N		-0.193	-0.587	0.202	0.338
Y	Adams JB et al.2011	0.101	-0.293	0.494	0.616
Y	Al-Farsi et al.2013	-0.757	-1.207	-0.308	0.001
Y	Ali et al.2011	-0.441	-0.880	-0.001	0.049
Y	Altun et al. 2018	-3.538	-4.152	-2.925	0.000
Y	Bala KA et al.2016	-1.324	-1.993	-0.656	0.000
Y	Chen et al. 2016	0.138	-0.197	0.473	0.419
Y	Parellada et al.2012	-0.126	-0.593	0.341	0.598
Y	Sun et al. 2016	0.143	-0.365	0.652	0.581
Y	Tu et al.2013	-0.564	-1.074	-0.055	0.030
Y		-0.687	-1.318	-0.055	0.033

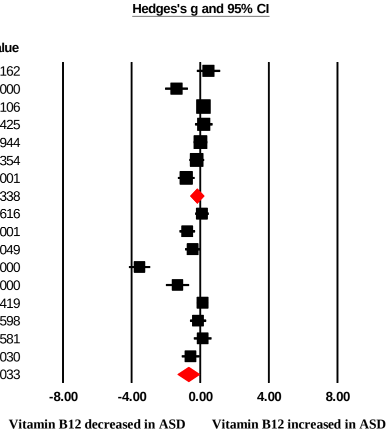

Forrest plot showing pooled results comparing vitamin B9 levels between ASD patients and HC subjects stratified by source of sampling (plasma heterogeneity: Q = 10.578, P = 0.032, I^2 = 62.187, 5 studies; and serum heterogeneity: Q = 164.768, P < 0.001, I^2 = 93.931, 11 studies), age matching (Y heterogeneity: Q = 172.258, P < 0.001, I^2 = 92.453, 14 studies) and gender matching (N heterogeneity: Q = 33.251, P < 0.001, I^2 = 81.956, 7 studies; and Y heterogeneity: Q = 132.385, P < 0.001, I^2 = 93.957, 9 studies) between ASD patients and HC subjects. The sizes of the squares are proportional to study weights.

eFigure 2

F

Vitamin D

Group by Source	Study name	Hedges's g	Lower limit	Upper limit	p-Value
serum	Altun et al. 2018	-2.656	-3.181	-2.130	0.000
serum	Arastoo et al.2018	-0.975	-1.496	-0.454	0.000
serum	Bala KA et al.2016	-1.573	-2.265	-0.881	0.000
serum	Bener et al.2014	-0.385	-0.560	-0.210	0.000
serum	Bicíková et al. 2019	-0.221	-0.645	0.202	0.305
serum	Chen et al. 2016	-0.574	-0.915	-0.233	0.001
serum	Coskun et al.2016	0.571	0.263	0.879	0.000
serum	Feng J et al.2016	-0.220	-0.397	-0.043	0.015
serum	Garipardic et al.2017	-1.775	-2.478	-1.071	0.000
serum	Gong et al.2013	-0.643	-1.050	-0.236	0.002
serum	Guo et al. 2018	-0.305	-0.537	-0.073	0.010
serum	Guo et al. 2019	-0.232	-0.409	-0.056	0.010
serum	Meguid et al.2010	-0.776	-1.169	-0.382	0.000
serum	Mostafa et al.2012	-0.782	-1.246	-0.318	0.001
serum	Saad et al. 2016	-2.686	-3.049	-2.323	0.000
serum	Saad et al.2017	-1.318	-1.862	-0.774	0.000
serum		-0.877	-1.236	-0.518	0.000

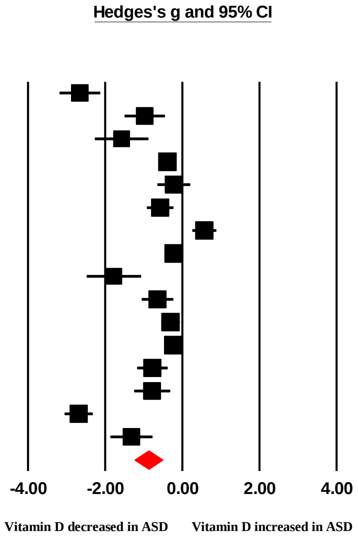

Vitamin D

Group by Gender matching	Study name	Hedges's g	Lower limit	Upper limit	p-Value
N	Bener et al.2014	-0.385	-0.560	-0.210	0.000
N	Coskun et al.2016	0.571	0.263	0.879	0.000
N	Garipardic et al.2017	-1.775	-2.478	-1.071	0.000
N	Guo et al. 2018	-0.305	-0.537	-0.073	0.010
N	Guo et al. 2019	-0.232	-0.409	-0.056	0.010
N	Meguid et al.2010	-0.776	-1.169	-0.382	0.000
N		-0.395	-0.751	-0.038	0.030
Y	Adams JB et al.2011	0.154	-0.240	0.547	0.445
Y	Altun et al. 2018	-2.656	-3.181	-2.130	0.000
Y	Arastoo et al.2018	-0.975	-1.496	-0.454	0.000
Y	Bala KA et al.2016	-1.573	-2.265	-0.881	0.000
Y	Bi?íková et al. 2019	-0.221	-0.645	0.202	0.305
Y	Chen et al. 2016	-0.574	-0.915	-0.233	0.001
Y	El-Ansary et al.2018	-1.947	-2.582	-1.311	0.000
Y	Feng J et al.2016	-0.220	-0.397	-0.043	0.015
Y	Gong et al.2013	-0.643	-1.050	-0.236	0.002
Y	Mostafa et al.2012	-0.782	-1.246	-0.318	0.001
Y	Saad et al. 2016	-2.686	-3.049	-2.323	0.000
Y	Saad et al.2017	-1.318	-1.862	-0.774	0.000
Y		-1.106	-1.650	-0.563	0.000

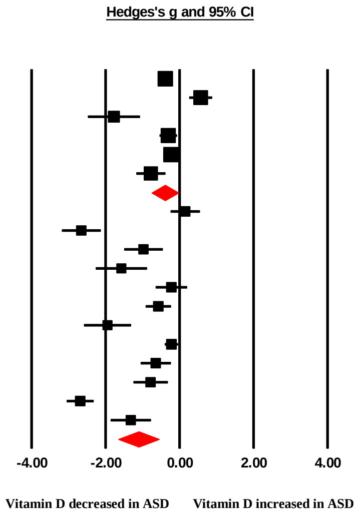

Forrest plot showing pooled results comparing serum vitamin D levels (heterogeneity: Q = 312.387, P < 0.001, I^2 = 95.198, 15 studies) and gender matching (N heterogeneity: Q = 54.444, P < 0.001, I^2 = 90.816, 6 studies; and Y heterogeneity: Q = 245.413, P < 0.001, I^2 = 95.518, 12 studies) between ASD patients and HC subjects. The sizes of the squares are proportional to study weights.

eFigure 2

GSH

G

Group by source	Study name	Hedges's g	Lower limit	Upper limit	p-Value
plasma	Adams et al.2011	-1.341	-1.776	-0.906	0.000
plasma	Al-Gadani et al.2009	-0.843	-1.365	-0.321	0.002
plasma	El-Ansary etl 2017	-1.380	-1.966	-0.793	0.000
plasma	Geier et al.2009	-1.539	-1.940	-1.138	0.000
plasma	James et al.2006	-1.107	-1.446	-0.768	0.000
plasma	James et al. 2009	-2.021	-2.550	-1.492	0.000
plasma	Kondolot et al.2016	0.032	-0.357	0.421	0.872
plasma	Melnyk et al.2012	-0.538	-0.950	-0.125	0.011
plasma	Oshodi et al. 2017	-0.702	-1.219	-0.185	0.008
plasma	Parellada et al.2012	0.012	-0.462	0.485	0.961
plasma	Rose et al.2012	-1.806	-2.325	-1.286	0.000
plasma	Suh et al.2008	-0.174	-0.850	0.502	0.613
plasma		-0.951	-1.336	-0.565	0.000

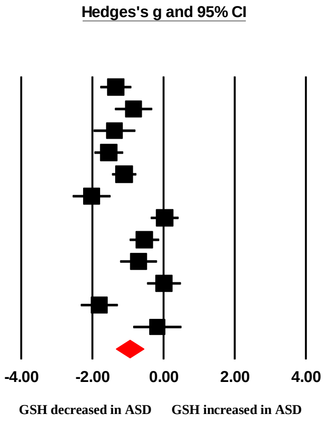

GSH

Group by Age matching	Study name	Hedges's g	Lower limit	Upper limit	p-Value
N	James et al.2006	-1.107	-1.446	-0.768	0.000
N	Rose et al.2012	-1.806	-2.325	-1.286	0.000
N	Suh et al.2008	-0.174	-0.850	0.502	0.613
N		-1.059	-1.826	-0.291	0.007
Y	Adams et al.2011	-1.341	-1.776	-0.906	0.000
Y	Al-Gadani et al.2009	-0.843	-1.365	-0.321	0.002
Y	El-Ansary etl 2017	-1.380	-1.966	-0.793	0.000
Y	Geier et al.2009	-1.539	-1.940	-1.138	0.000
Y	Han et al.2015	-0.455	-0.849	-0.061	0.024
Y	Hodgson et al. 2014	-0.541	-1.076	-0.005	0.048
Y	James et al. 2009	-2.021	-2.550	-1.492	0.000
Y	Kondolot et al.2016	0.032	-0.357	0.421	0.872
Y	Melnyk et al.2012	-0.538	-0.950	-0.125	0.011
Y	Oshodi et al. 2017	-0.702	-1.219	-0.185	0.008
Y	Parellada et al.2012	0.012	-0.462	0.485	0.961
Y		-0.839	-1.227	-0.450	0.000

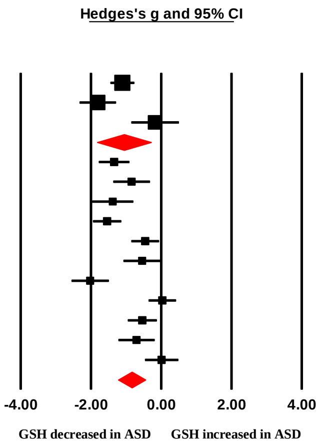

GSH

Group by Gender matching	Study name	Hedges's g	Lower limit	Upper limit	p-Value
N	Al-Gadani et al.2009	-0.843	-1.365	-0.321	0.002
N	El-Ansary etl 2017	-1.380	-1.966	-0.793	0.000
N	Geier et al.2009	-1.539	-1.940	-1.138	0.000
N	James et al.2006	-1.107	-1.446	-0.768	0.000
N	James et al. 2009	-2.021	-2.550	-1.492	0.000
N	Melnyk et al.2012	-0.538	-0.950	-0.125	0.011
N	Oshodi et al. 2017	-0.702	-1.219	-0.185	0.008
N	Rose et al.2012	-1.806	-2.325	-1.286	0.000
N	Suh et al.2008	-0.174	-0.850	0.502	0.613
N		-1.133	-1.498	-0.768	0.000
Y	Adams et al.2011	-1.341	-1.776	-0.906	0.000
Y	Han et al.2015	-0.455	-0.849	-0.061	0.024
Y	Hodgson et al. 2014	-0.541	-1.076	-0.005	0.048
Y	Kondolot et al.2016	0.032	-0.357	0.421	0.872
Y	Parellada et al.2012	0.012	-0.462	0.485	0.961
Y		-0.457	-0.956	0.043	0.073

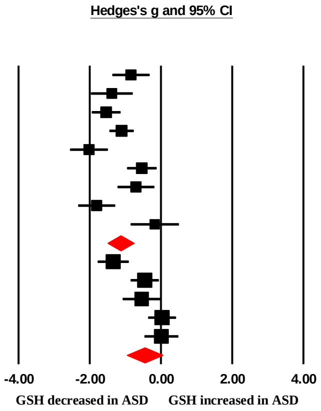

Forrest plot showing pooled results comparing plasma GSH levels (heterogeneity: Q = 90.649, P < 0.001, I^2 = 87.865, 12 studies), age matching (N heterogeneity: Q = 14.173, P = 0.001, I^2 = 85.889, 3 studies; and Y heterogeneity: Q = 77.621, P < 0.001, I^2 = 87.117, 11 studies) and gender matching (N heterogeneity: Q = 41.470, P < 0.001, I^2 = 80.709, 9 studies; and Y heterogeneity: Q = 25.738, P < 0.001, I^2 = 84.459, 5 studies) between ASD patients and HC subjects. The sizes of the squares are proportional to study weights.

eFigure 3

Meta-regression for Homocysteine

A

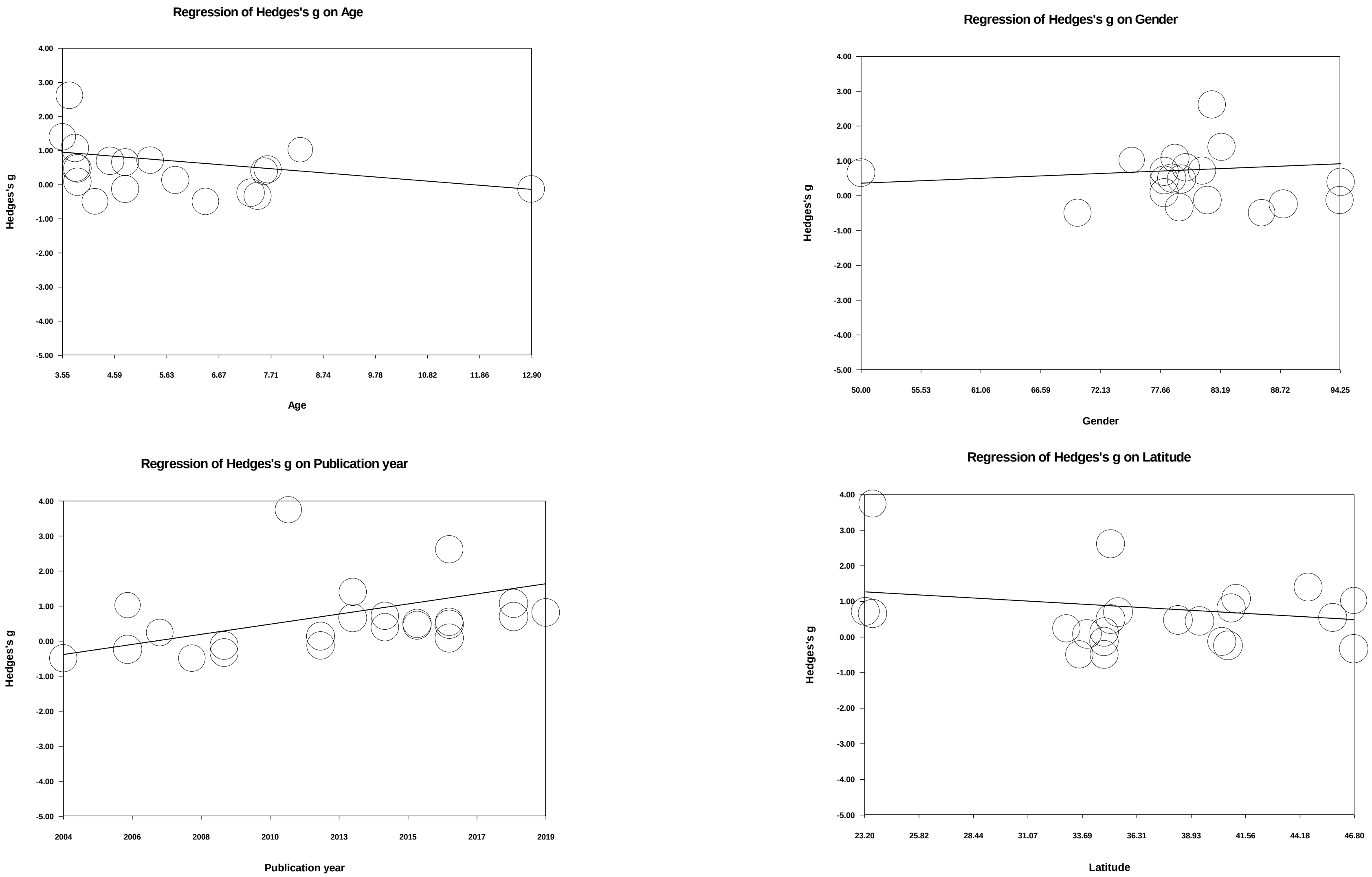

Association between age (Regression coefficient[SE]: -0.1168 [0.0871], 95%CI: -0.2875 to 0.0539, p = 0.1800), gender (Regression coefficient[SE]: 0.0126 [0.0225], 95%CI: -0.0314 to 0.0567, p = 0.5744), publication year (Regression coefficient[SE]: 0.1346 [0.0425], 95%CI: 0.0513 to 0. 2179, p = 0.0015), latitude (Regression coefficient [SE]: -0.0330 [0.0306], 95% CI: -0.0.929 to 0.0270, p = 0.2809) and effective size (Hedges'sg) for homocysteine.

eFigure 3

Meta-regression for Methionine

B

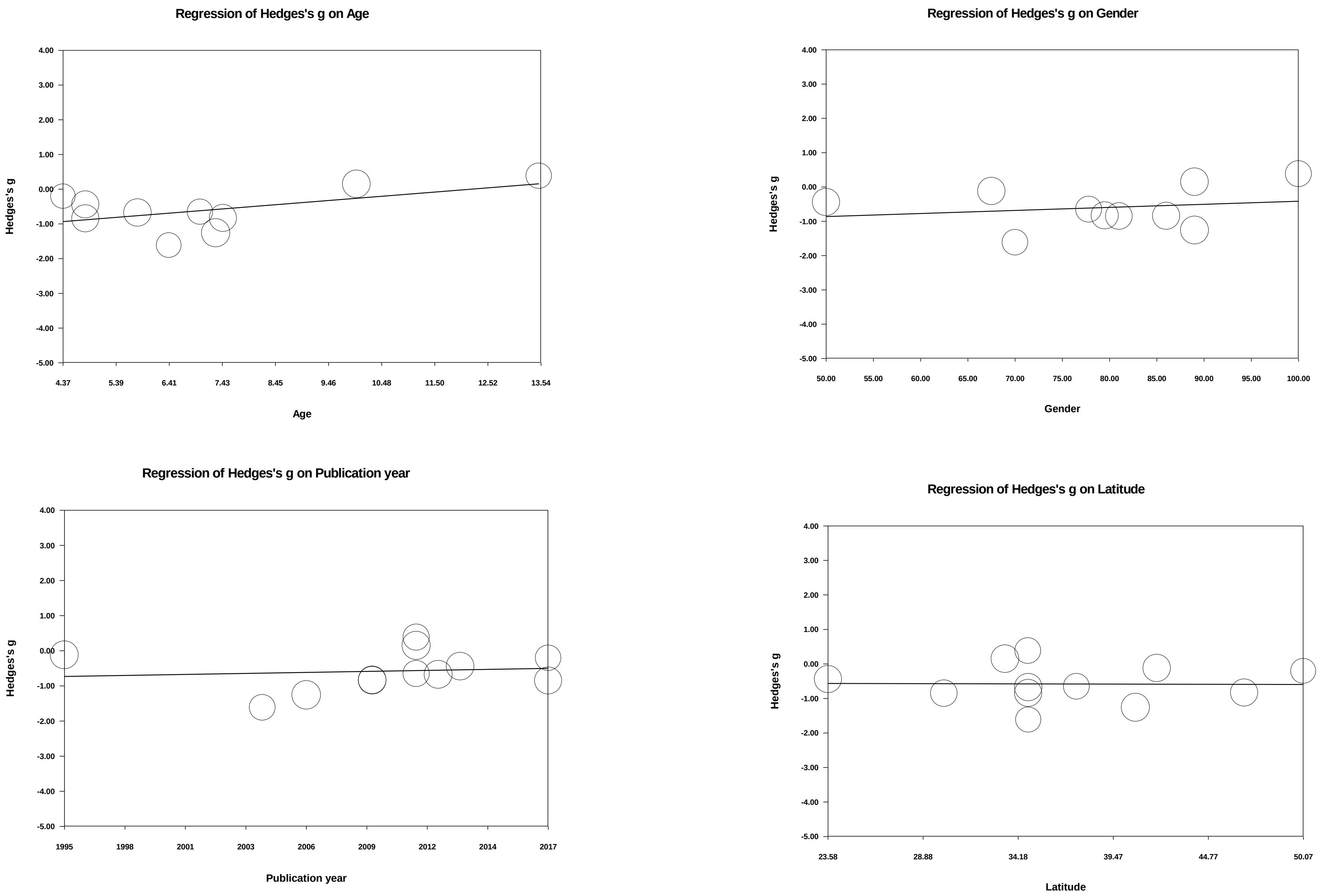

Association between age (Regression coefficient[SE]: 0.1191 [0.0640], 95%CI: -0.0064 to 0.2446, p = 0.0628), gender (Regression coefficient[SE]: 0.0089 [0.0152], 95%CI: -0.0208 to 0.0387, p = 0.5560), publication year (Regression coefficient[SE]: 0.0104 [0.0295], 95%CI: -0.0473to 0.0682, p = 0.7232), latitude (Regression coefficient[SE]: -0.0011 [0.0245], 95%CI: -0.0492 to 0.0470, p = 0.9635) and effective size (Hedges’sg) for methionine.

eFigure 3

Meta-regression for Cysteine

C

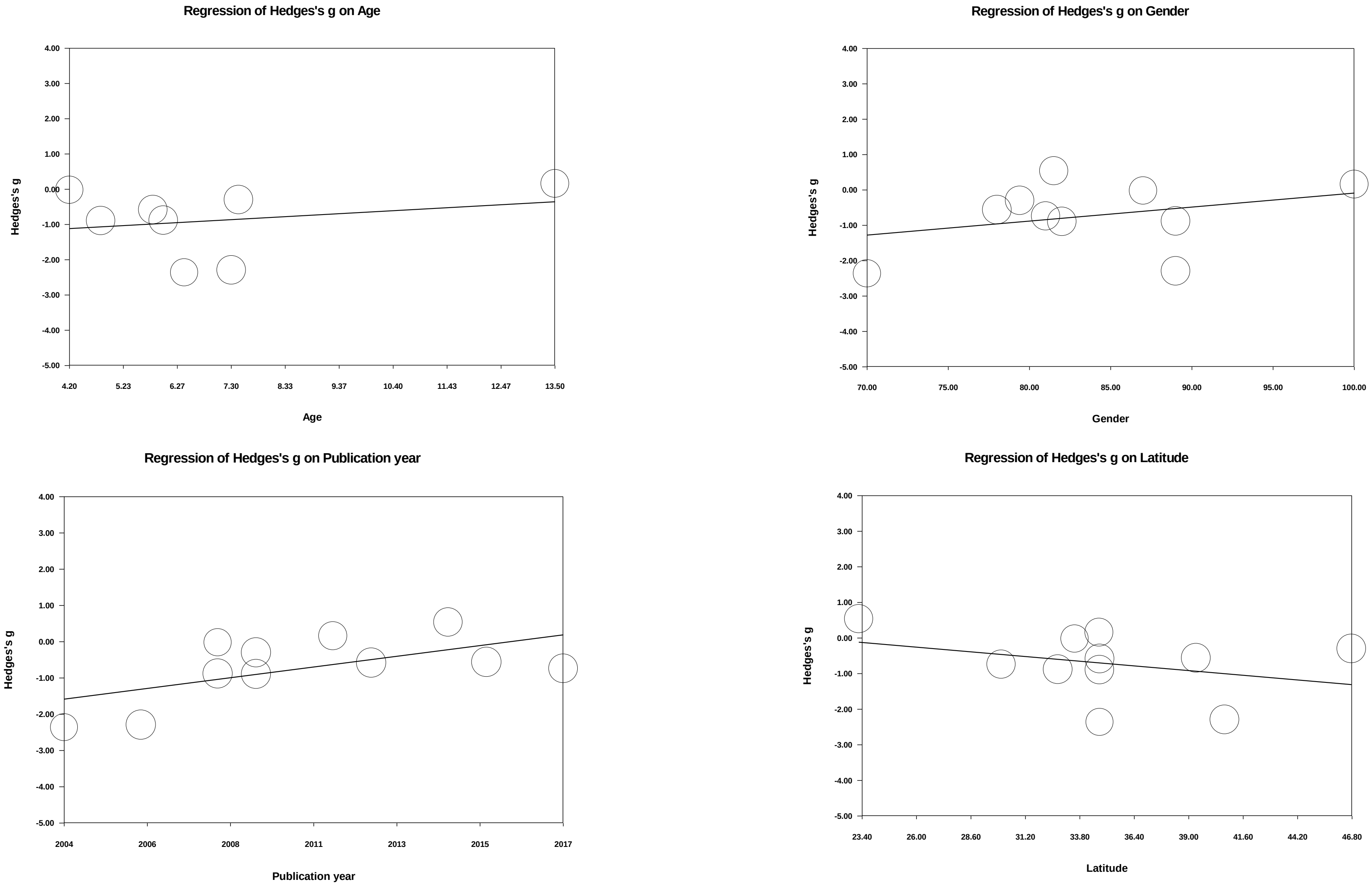

Association between age (Regression coefficient[SE]: 0.0457 [0.1188], 95%CI: -0.1871 to 0.2785, p = 0.7005), gender (Regression coefficient[SE]: 0.0396 [0.0395], 95%CI: -0.0377 to 0.1169, p = 0.3155), publication year (Regression coefficient[SE]: 0.1363 [0.0579], 95%CI: 0.0229 to 0.2498, p = 0.0185), latitude (Regression coefficient[SE]: -0.0506 [0.0440], 95%CI: -0.1369 to 0.0356, p = 0.2500) and effective size (Hedges'sg) for cysteine.

eFigure 3

Meta-regression for Vitamin B9

D

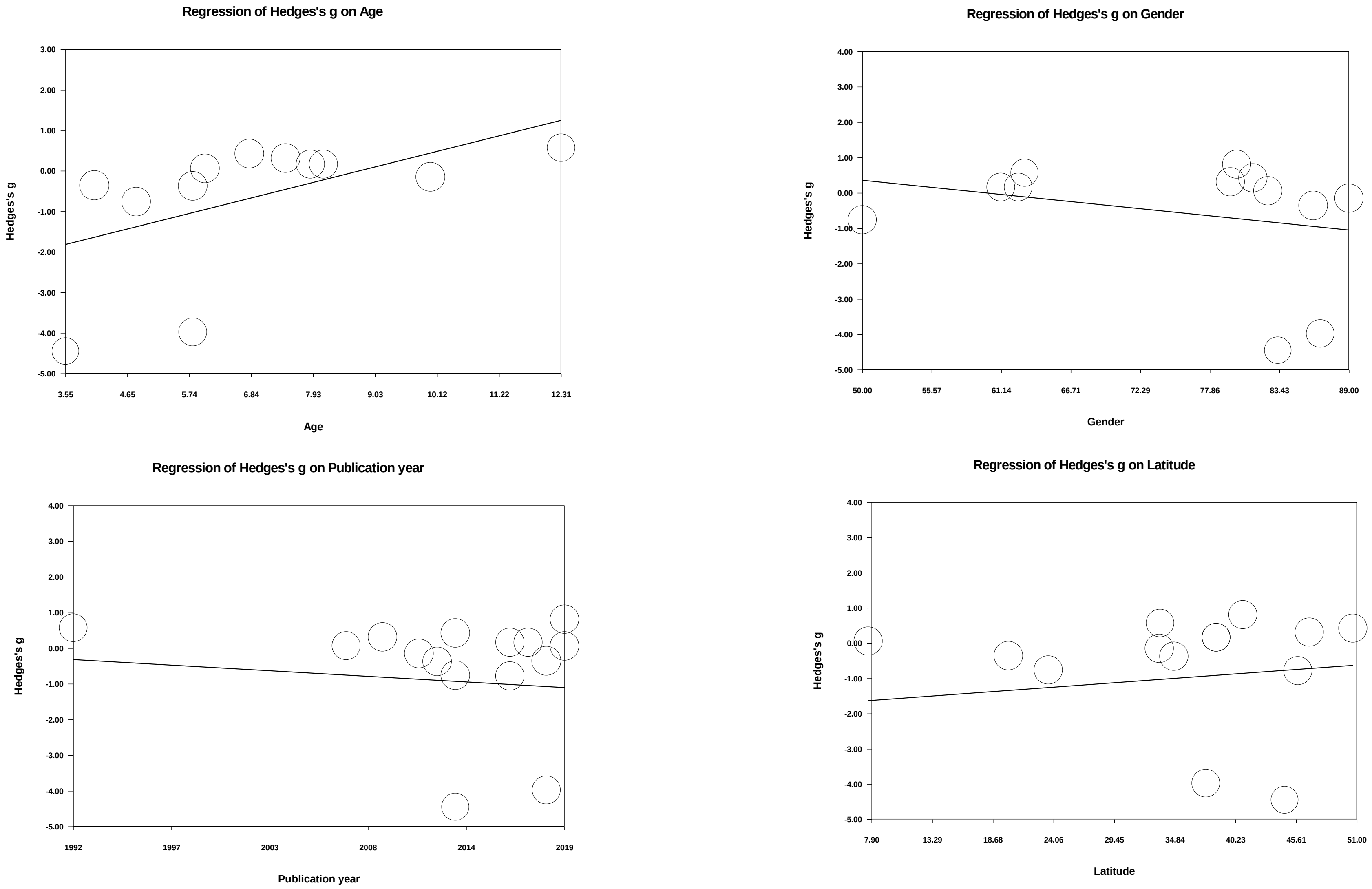

Association between age (Regression coefficient[SE]: 0.3497 [0.1331], 95%CI: 0.0889 to 0.6105, p = 0.0086), gender (Regression coefficient[SE]: -0.0361 [0.0281], 95%CI: -0.0911 to 0.0189, p = 0.1985), publication year (Regression coefficient[SE]: -0.0291 [0.0511], 95%CI: -0.1292 to 0.0710, p = 0.5688), latitude (Regression coefficient[SE]: 0.0233 [0.0311], 95%CI: -0.0376 to 0.0842, p = 0.4532) and effective size (Hedges'sg) for vitamin B9.

eFigure 3

Meta-regression for Vitamin B12

E

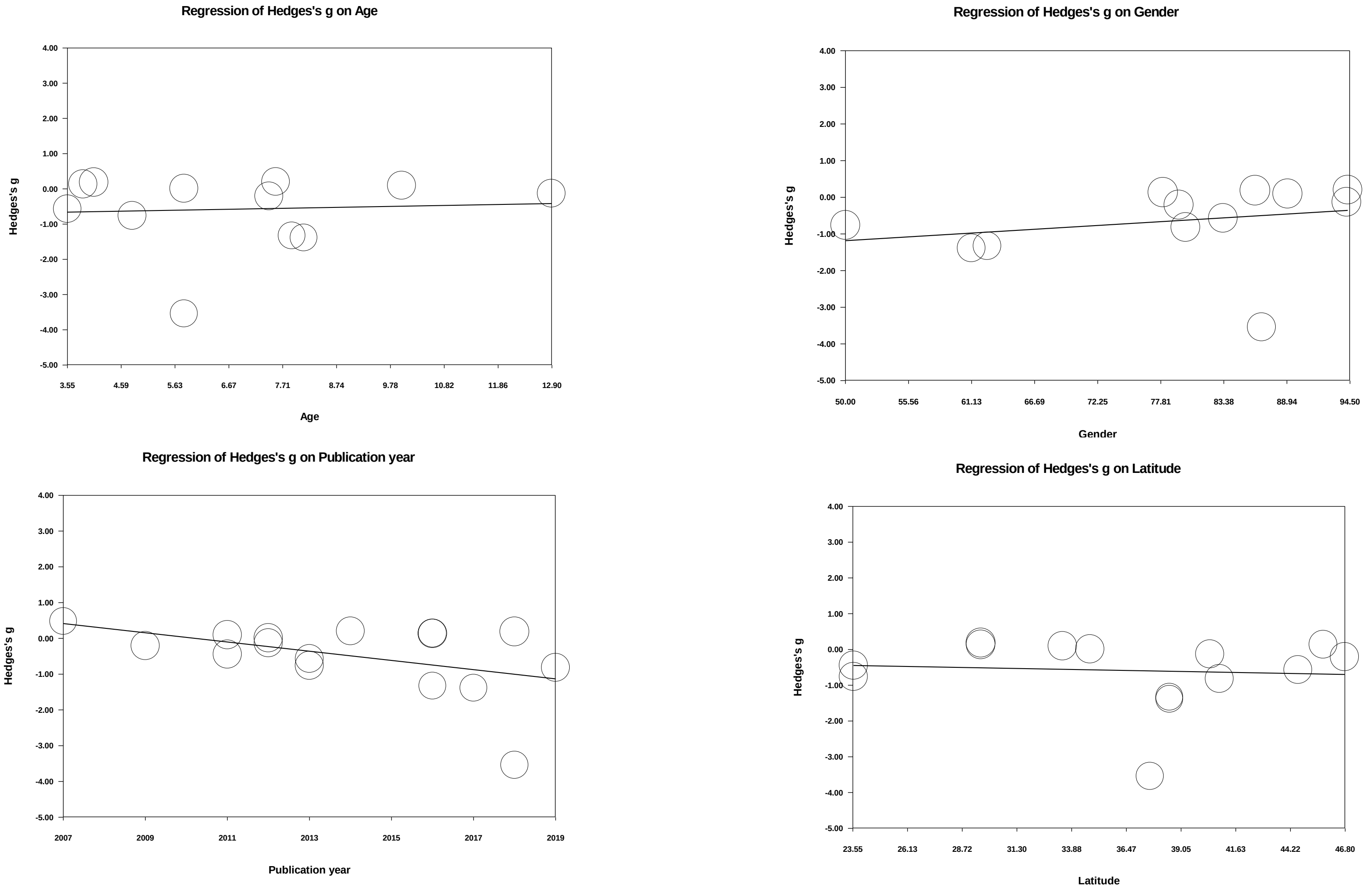

Association between age (Regression coefficient[SE]: 0.0260 [0.0977], 95%CI: -0.1656 to 0.2175, p = 0.7905), gender (Regression coefficient[SE]: 0.0187 [0.0187], 95%CI: -0.0180 to 0.0554, p = 0.3184), publication year (Regression coefficient[SE]: -0.1290 [0.0612], 95%CI: -0.2489 to -0.0091, p = 0.0350), latitude (Regression coefficient[SE]: -0.0108 [0.0300], 95%CI: -0.0696 to 0.0480, p = 0.7196) and effective size (Hedges’sg) for vitamin B12.

eFigure 3

Meta-regression for Vitamin D

F

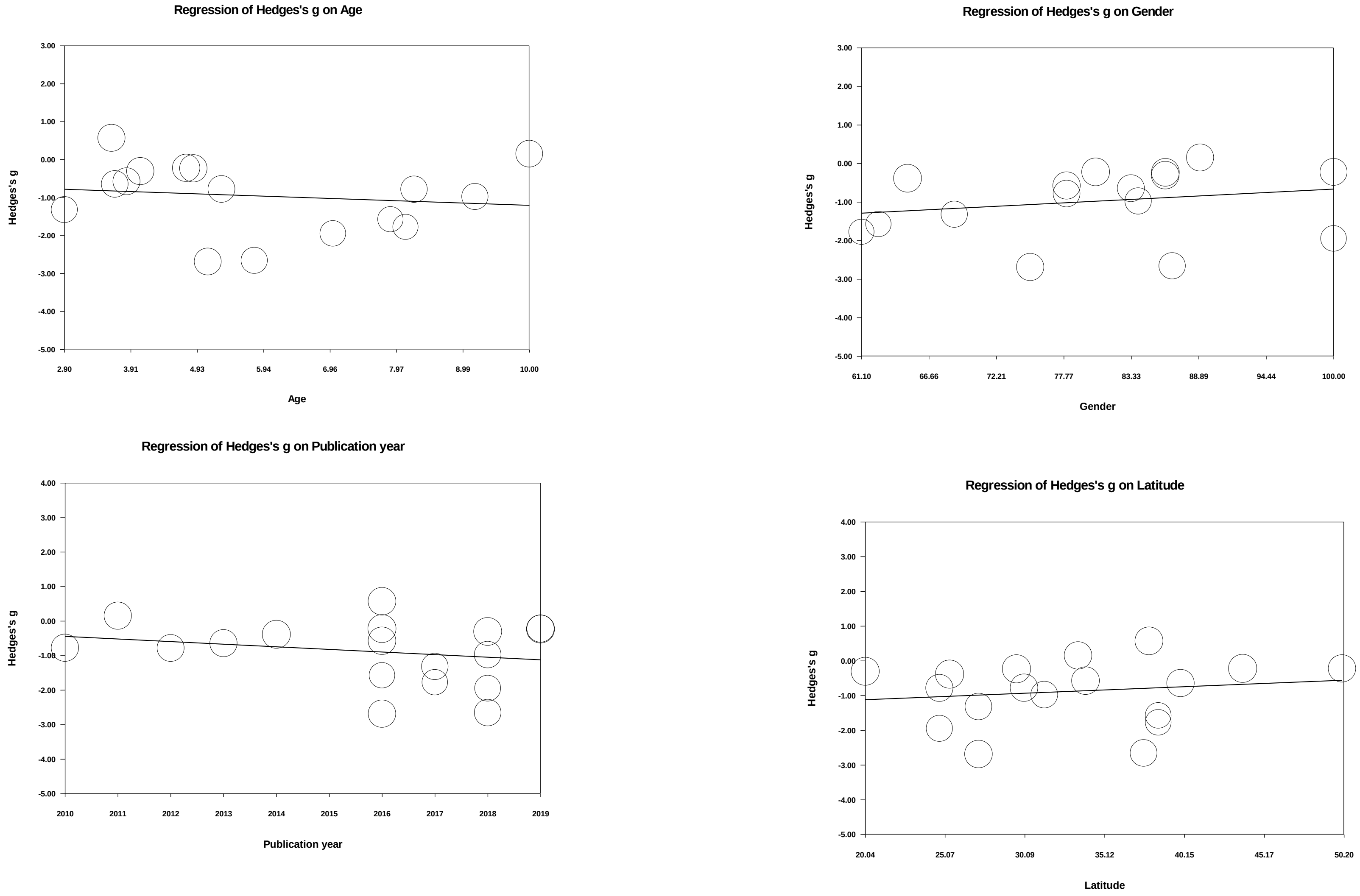

Association between age (Regression coefficient [SE]: -0.0597 [0.0986], 95%CI: -0.2530 to 0.1337, p = 0.5453), gender (Regression coefficient [SE]: 0.0161 [0.0174], 95%CI: -0.0180 to 0.0501, p = 0.3559), publication year (Regression coefficient[SE]: -0.0754 [0.0697], 95%CI: -0.2121 to 0.0613, p = 0.2794), latitude (Regression coefficient[SE]: 0.0187 [0.0245], 95%CI: -0.0293 to 0.0666, p = 0.4460) and effective size (Hedges'sg) for vitamin D.

eFigure 3

Meta-regression for GSH

G

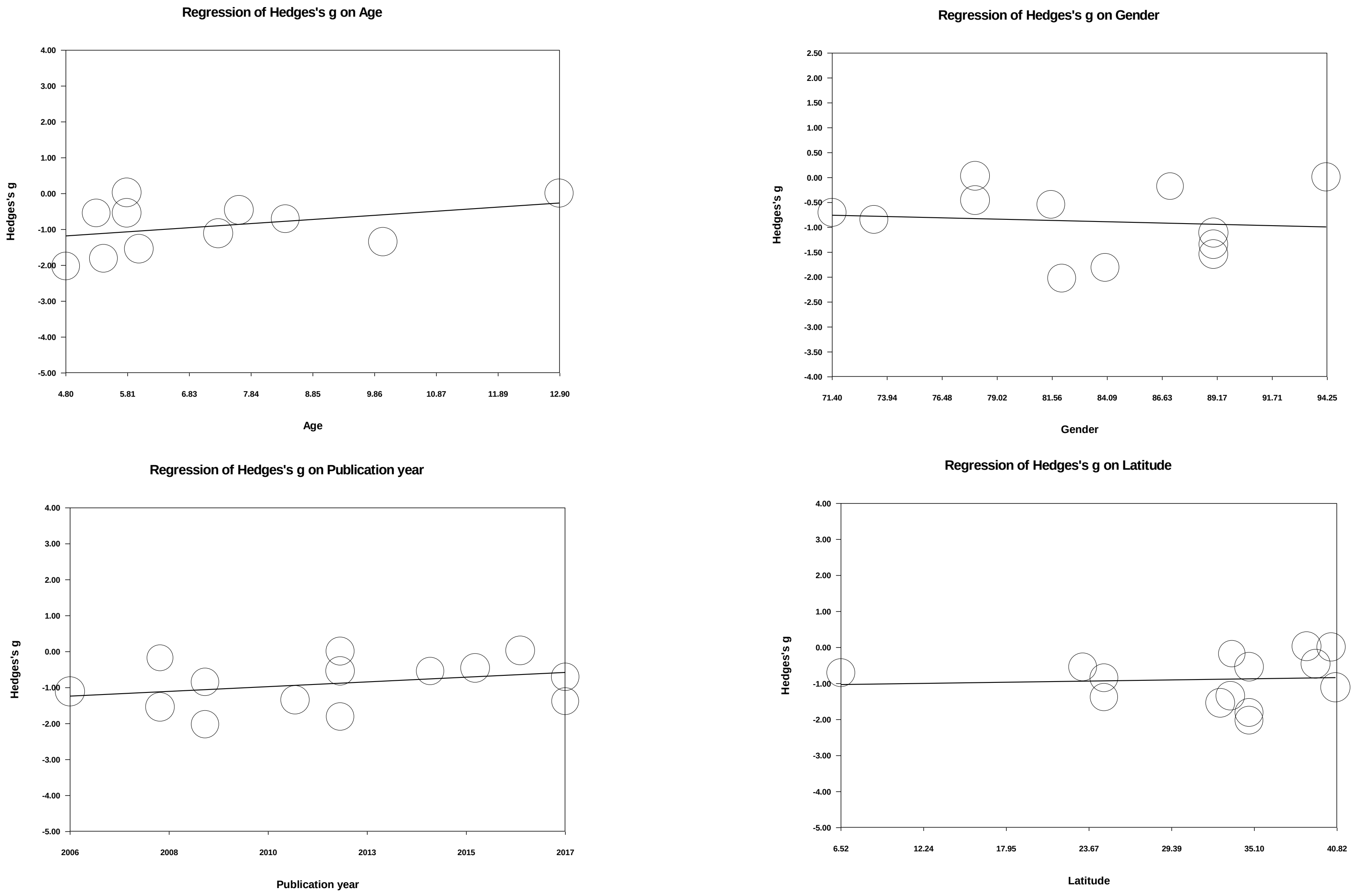

Association between age (Regression coefficient [SE]: 0.1134 [0.0872], 95%CI: -0.0575 to 0.2844, p = 0.1934), gender (Regression coefficient[SE]: -0.0103 [0.0301], 95%CI: -0.0694 to 0.0488, p = 0.7320), publication year (Regression coefficient[SE]: 0.0598 [0.0496], 95%CI: -0.0321 to 0.1517, p = 0.2021), latitude (Regression coefficient[SE]: 0.0056 [0.0201], 95%CI: -0.0339 to 0.0451, p = 0.7806) and effective size (Hedges'sg) for GSH.

eFigure 4

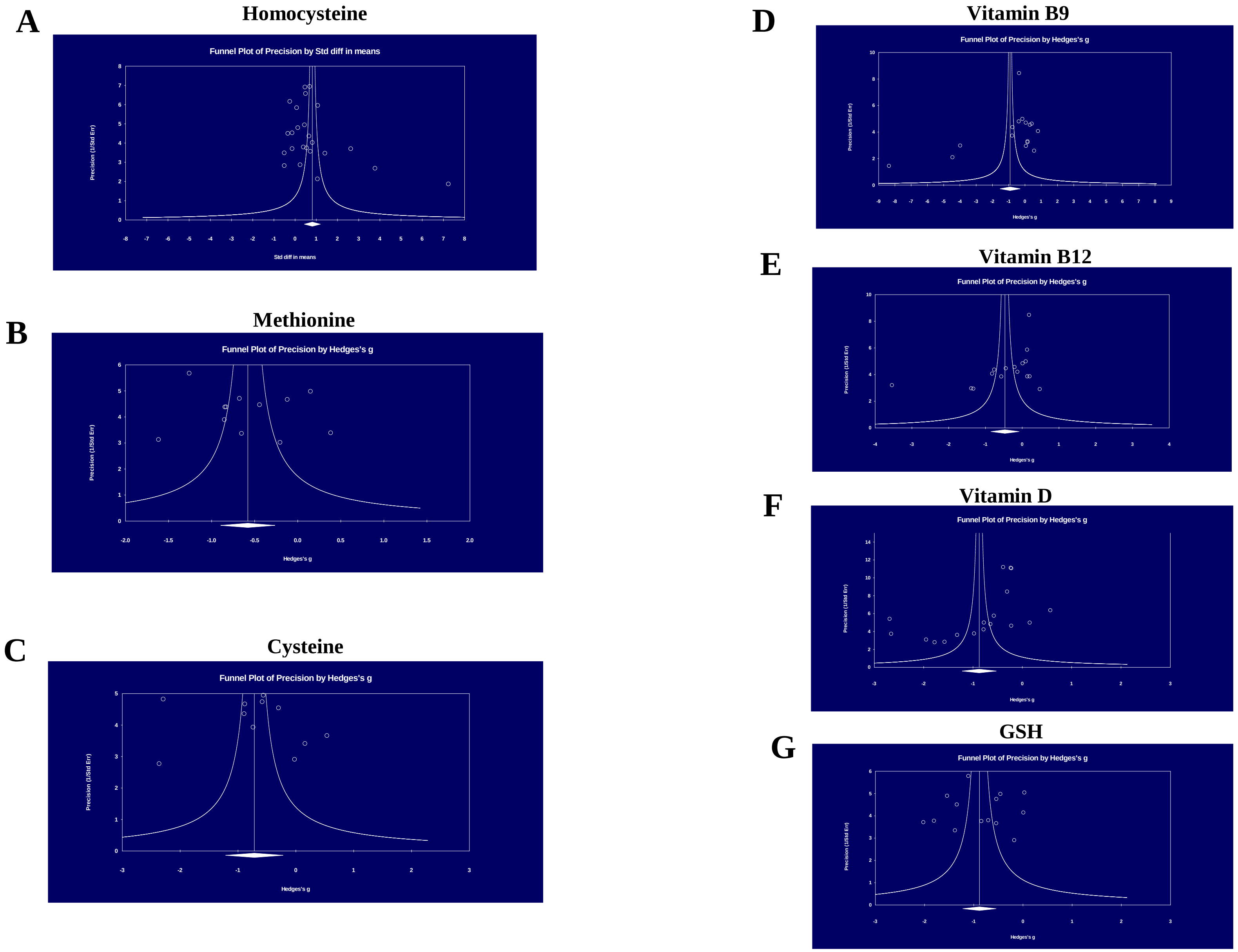

Funnel plots for studies analyzing blood homocysteine, methionine, cysteine, vitamin B9, vitamin B12, vitamin D and GSH levels

eReference：

1. Al-Yafee YA, Al-Ayadhi LY, Haq SH, El-Ansary AK. Novel metabolic biomarkers related to sulfur-dependent detoxification pathways in autistic patients of Saudi Arabia. *BMC Neurol.* 2011; 11:139.
2. Al-Gadani Y, El-Ansary A, Attas O, Al-Ayadhi L. Metabolic biomarkers related to oxidative stress and antioxidant status in Saudi autistic children. *Clin Biochem.* 2009;42(10-11):1032-1040.
3. Rose S, Melnyk S, Trusty TA, et al. Intracellular and extracellular redox status and free radical generation in primary immune cells from children with autism. *Autism Res Treat.* 2012; 2012:986519.
4. El-Ansary A, Al-Daihan S, Al-Dbass A, Al-Ayadhi L. Measurement of selected ions related to oxidative stress and energy metabolism in Saudi autistic children. *Clin Biochem.* 2010;43(1-2):63-70.
5. El-Ansary A, Bjorklund G, Chirumbolo S, Alnakhli OM. Predictive value of selected biomarkers related to metabolism and oxidative stress in children with autism spectrum disorder. *Metab Brain Dis.* 2017;32(4):1209-1221.
6. Essa MM, Guillemin GJ, Waly MI, et al. Increased markers of oxidative stress in autistic children of the Oman. *Biol Trace Elem Res.* 2012;147(1-3):25-27.
7. Grayaa S, Zerbinati C, Messedi M, et al. Plasma oxysterol profiling in children reveals 24-hydroxycholesterol as a potential marker for Autism Spectrum Disorders. *Biochimie.* 2018; 153:80-85.
8. James SJ, Cutler P, Melnyk S, et al. Metabolic biomarkers of increased oxidative stress and impaired methylation capacity in children with autism. *Am J Clin Nutr.* 2004;80(6):1611-1617.
9. El-Ansary A. Data of multiple regressions analysis between selected biomarkers related to glutamate excitotoxicity and oxidative stress in Saudi autistic patients. *Data Brief.* 2016; 7:111-116.
10. Meguid NA, Dardir AA, Abdel-Raouf ER, Hashish A. Evaluation of oxidative stress in autism: defective antioxidant enzymes and increased lipid peroxidation. *Biol Trace Elem Res.* 2011;143(1):58-65.
11. Mostafa GA, El-Hadidi ES, Hewedi DH, Abdou MM. Oxidative stress in Egyptian children with autism: relation to autoimmunity. *J Neuroimmunol.* 2010;219(1-2):114-118.
12. Oshodi Y, Ojewunmi O, Oshodi TA, et al. Oxidative stress markers and genetic polymorphisms of glutathione S-transferase T1, M1, and P1 in a subset of children with autism spectrum disorder in Lagos, Nigeria. *Niger J Clin Pract.* 2017;20(9):1161-1167.
13. Yenkoyan K, Harutyunyan H, Harutyunyan A. A certain role of SOD/CAT imbalance in pathogenesis of autism spectrum disorders. *Free Radic Biol Med.* 2018; 123:85-95.
14. Yui K, Tanuma N, Yamada H, Kawasaki Y. Reduced endogenous urinary total antioxidant power and its relation of plasma antioxidant activity of superoxide dismutase in individuals with autism spectrum disorder. *Int J Dev Neurosci.* 2017; 60:70-77.
15. Yui K, Tanuma N, Yamada H, Kawasaki Y. Decreased total antioxidant capacity has a larger effect size than increased oxidant levels in urine in individuals with autism spectrum disorder. *Environ Sci Pollut Res Int.* 2017;24(10):9635-9644.
16. Adams JB, George F, Audhya T. Abnormally high plasma levels of vitamin B6 in children with autism not taking supplements compared to controls not taking supplements. *J Altern Complement Med.* 2006;12(1):59-63.
17. Adams JB, Audhya T, McDonough-Means S, et al. Nutritional and metabolic status of children with autism vs. neurotypical children, and the association with autism severity. *Nutr Metab (Lond).* 2011;8(1):34.
18. Adams M, Lucock M, Stuart J, Fardell S, Baker K, Ng X. Preliminary evidence for involvement of the folate gene polymorphism 19bp deletion-DHFR in occurrence of autism. *Neurosci Lett.* 2007;422(1):24-29.

19. D'Eufemia P, Finocchiaro R, Celli M, Viozzi L, Monteleone D, Giardini O. Low serum tryptophan to large neutral amino acids ratio in idiopathic infantile autism. *Biomed Pharmacother.* 1995;49(6):288-292.
20. Eto I, Bandy MD, Butterworth CE, Jr. Plasma and urinary levels of biopterin, neopterin, and related pterins and plasma levels of folate in infantile autism. *J Autism Dev Disord.* 1992;22(2):295-308.
21. Fatemi SH, Stary JM, Egan EA. Reduced blood levels of reelin as a vulnerability factor in pathophysiology of autistic disorder. *Cell Mol Neurobiol.* 2002;22(2):139-152.
22. Geier DA, Kern JK, Garver CR, Adams JB, Audhya T, Geier MR. A prospective study of transsulfuration biomarkers in autistic disorders. *Neurochem Res.* 2009;34(2):386-393.
23. James SJ, Melnyk S, Jernigan S, et al. Metabolic endophenotype and related genotypes are associated with oxidative stress in children with autism. *Am J Med Genet B Neuropsychiatr Genet.* 2006;141B(8):947-956.
24. Meguid NA, Hashish AF, Anwar M, Sidhom G. Reduced serum levels of 25-hydroxy and 1,25-dihydroxy vitamin D in Egyptian children with autism. *J Altern Complement Med.* 2010;16(6):641-645.
25. Melnyk S, Fuchs GJ, Schulz E, et al. Metabolic imbalance associated with methylation dysregulation and oxidative damage in children with autism. *J Autism Dev Disord.* 2012;42(3):367-377.
26. Pasca SP, Nemes B, Vlase L, et al. High levels of homocysteine and low serum paraoxonase 1 arylesterase activity in children with autism. *Life Sci.* 2006;78(19):2244-2248.
27. Pasca SP, Dronca E, Kaucsar T, et al. One carbon metabolism disturbance and the C677T MTHFR gene polymorphism in children with autism spectrum disorders. *J Cell Mol Med.* 2009;13(10):4229-4238.
28. Sogut S, Zoroglu SS, Ozyurt H, et al. Changes in nitric oxide levels and antioxidant enzyme activities may have a role in the pathophysiological mechanisms involved in autism. *Clin Chim Acta.* 2003;331(1-2):111-117.
29. Suh, J. H.; Walsh, W. J.; McGinnis, W. R.; Lewis, A.; Ames, B. N. Altered sulfur amino acid metabolism in immune cells of children diagnosed with autism.*Am J Biochem Biotechnol* 4:105–113; 2008.
30. Sweeten TL, Posey DJ, Shankar S, McDougle CJ. High nitric oxide production in autistic disorder: a possible role for interferon-gamma. *Biol Psychiatry*. 2004;55(4):434-437.
31. Vergani, L.; Lanza, C.; Rivaro, P.; Abelmoschi, M. L.; Genti, S.; Veneselli, E.; Minniti, G.; Grasselli, E.; Canesi, L.; Voci, A. Metals, metallothioneins and oxidative stress in blood of autistic children. *Res Autism Spectrum Disord* 5:286–293; 2011.
32. Yorbik O, Sayal A, Akay C, Akbiyik DI, Sohmen T. Investigation of antioxidant enzymes in children with autistic disorder. *Prostaglandins Leukot Essent Fatty Acids.* 2002;67(5):341-343.
33. Bala KA, Dogan M, Kaba S, Mutluer T, Aslan O, Dogan SZ. Hormone disorder and vitamin deficiency in attention deficit hyperactivity disorder (ADHD) and autism spectrum disorders (ASDs). *J Pediatr Endocrinol Metab.* 2016;29(9):1077-1082.
34. Chauhan A, Chauhan V, Brown WT, Cohen I. Oxidative stress in autism: increased lipid peroxidation and reduced serum levels of ceruloplasmin and transferrin--the antioxidant proteins. *Life Sci.* 2004;75(21):2539-2549.
35. Skalny AV, Simashkova NV, Klyushnik TP, et al. Assessment of serum trace elements and electrolytes in children with childhood and atypical autism. *J Trace Elem Med Biol.* 2017; 43:9-14.
36. Skalny AV, Simashkova NV, Skalnaya AA, et al. Assessment of gender and age effects on serum and hair trace element levels in children with autism spectrum disorder. *Metab Brain Dis.* 2017;32(5):1675-1684.
37. Yui K, Imataka G, Kawasak Y, Yamada H. Increased omega-3 polyunsaturated fatty acid/arachidonic acid ratios and upregulation of signaling mediator in individuals with autism spectrum disorders. *Life Sci.* 2016; 145:205-212.

38. Bener A, Khattab AO, Al-Dabbagh MM. Is high prevalence of Vitamin D deficiency evidence for autism disorder? In a highly endogamous population. *J Pediatr Neurosci*. 2014; 9:227-33.
39. Al-Farsi YM, Waly MI, Deth RC, et al. Low folate and vitamin B12 nourishment is common in Omani children with newly diagnosed autism. *Nutrition.* 2013;29(3):537-541.
40. Ali A, Waly MI, Al-Farsi YM, Essa MM, Al-Sharbati MM, Deth RC. Hyperhomocysteinemia among Omani autistic children: a case-control study. *Acta Biochim Pol.* 2011;58(4):547-551.
41. Arastoo AA, Khojastehkia H, Rahimi Z, et al. Evaluation of serum 25-Hydroxy vitamin D levels in children with autism Spectrum disorder. *Ital J Pediatr.* 2018;44(1):150.
42. Bugajska J, Berska J, Wojtyto T, Bik-Multanowski M, Sztefko K. The amino acid profile in blood plasma of young boys with autism. *Psychiatr Pol.* 2017;51(2):359-368.
43. Cai J, Ding L, Zhang JS, Xue J, Wang LZ. Elevated plasma levels of glutamate in children with autism spectrum disorders. *Neuroreport.* 2016;27(4):272-276.
44. Cortelazzo A, De Felice C, Guerranti R, et al. Expression and oxidative modifications of plasma proteins in autism spectrum disorders: Interplay between inflammatory response and lipid peroxidation. *Proteomics Clin Appl.* 2016;10(11):1103-1112.
45. Coskun S, Simsek S, Camkurt MA, Cim A, Celik SB. Association of polymorphisms in the vitamin D receptor gene and serum 25-hydroxyvitamin D levels in children with autism spectrum disorder. *Gene.* 2016;588(2):109-114.
46. El-Ansary AK, Ben Bacha AG, Al-Ayadhi LY. Proinflammatory and proapoptotic markers in relation to mono and di-cations in plasma of autistic patients from Saudi Arabia. *J Neuroinflammation.* 2011; 8:142.
47. El-Ansary A, Cannell JJ, Bjorklund G, et al. In the search for reliable biomarkers for the early diagnosis of autism spectrum disorder: the role of vitamin D. *Metab Brain Dis.* 2018;33(3):917-931.
48. Feng J, Shan L, Du L, et al. Clinical improvement following vitamin D3 supplementation in Autism Spectrum Disorder. *Nutr Neurosci.* 2017;20(5):284-290.
49. Garipardic M, Dogan M, Bala KA, et al. Association of Attention Deficit Hyperactivity Disorder and Autism Spectrum Disorders with Mean Platelet Volume and Vitamin D. *Med Sci Monit.* 2017; 23:1378-1384.
50. Gong ZL, Luo CM, Wang L, et al. Serum 25-hydroxyvitamin D levels in Chinese children with autism spectrum disorders. *Neuroreport.* 2014;25(1):23-27.
51. Gunes S, Ekinci O, Celik T. Iron deficiency parameters in autism spectrum disorder: clinical correlates and associated factors. *Ital J Pediatr.* 2017;43(1):86.
52. Han Y, Xi QQ, Dai W, et al. Abnormal transsulfuration metabolism and reduced antioxidant capacity in Chinese children with autism spectrum disorders. *Int J Dev Neurosci.* 2015; 46:27-32.
53. Hassan MH, Desoky T, Sakhr HM, Gabra RH, Bakri AH. Possible Metabolic Alterations among Autistic Male Children: Clinical and Biochemical Approaches. *J Mol Neurosci.* 2019;67(2):204-216.
54. Kim EK, Neggers YH, Shin CS, Kim E, Kim EM. Alterations in lipid profile of autistic boys: a case control study. *Nutr Res.* 2010;30(4):255-260.
55. Kondolot M, Ozmert EN, Asci A, et al. Plasma phthalate and bisphenol a levels and oxidant-antioxidant status in autistic children. *Environ Toxicol Pharmacol.* 2016; 43:149-158.
56. Li SO, Wang JL, Bjorklund G, Zhao WN, Yin CH. Serum copper and zinc levels in individuals with autism spectrum disorders. *Neuroreport.* 2014;25(15):1216-1220.
57. Mostafa GA, Al-Ayadhi LY. Reduced serum concentrations of 25-hydroxy vitamin D in children with autism: relation to autoimmunity. *J Neuroinflammation.* 2012; 9:201.
58. Parellada M, Moreno C, Mac-Dowell K, et al. Plasma antioxidant capacity is reduced in Asperger syndrome. *J Psychiatr Res.* 2012;46(3):394-401.
59. Ramaekers VT, Quadros EV, Sequeira JM. Role of folate receptor autoantibodies in infantile autism. *Mol Psychiatry.* 2013;18(3):270-271.
60. Saad K, Zahran AM, Elsayh KI, et al. Frequency of Dendritic Cells and Their Expression of Costimulatory Molecules in Children with Autism Spectrum Disorders. *J*

Autism Dev Disord. 2017;47(9):2671-2678.
61. Tirouvanziam R, Obukhanych TV, Laval J, et al. Distinct plasma profile of polar neutral amino acids, leucine, and glutamate in children with Autism Spectrum Disorders. *J Autism Dev Disord.* 2012;42(5):827-836.
62. Tostes MH, Teixeira HC, Gattaz WF, Brandao MA, Raposo NR. Altered neurotrophin, neuropeptide, cytokines and nitric oxide levels in autism. *Pharmacopsychiatry.* 2012;45(6):241-243.
63. Tu WJ, Yin CH, Guo YQ, et al. Serum homocysteine concentrations in Chinese children with autism. *Clin Chem Lab Med.* 2013;51(2): e19-22.
64. Wang L, Jia J, Zhang J, Li K. Serum levels of SOD and risk of autism spectrum disorder: A case-control study. *Int J Dev Neurosci.* 2016; 51:12-16.
65. Yui K, Imataka G, Kawasaki Y, Yamada H. Down-regulation of a signaling mediator in association with lowered plasma arachidonic acid levels in individuals with autism spectrum disorders. *Neurosci Lett.* 2016; 610:223-228.
66. Zaki MM, Abdel-Al H, Al-Sawi M. Assessment of plasma amino acid profile in autism using cation-exchange chromatography with postcolumn derivatization by ninhydrin. *Turk J Med Sci.* 2017;47(1):260-267.
67. Zhang QB, Gao SJ, Zhao HX. Thioredoxin: a novel, independent diagnosis marker in children with autism. *Int J Dev Neurosci.* 2015; 40:92-96.
68. Zhou W, Li S. Decreased levels of serum retinoic acid in chinese children with autism spectrum disorder. *Psychiatry Res.* 2018; 269:469-473.
69. Shimmura C, Suda S, Tsuchiya KJ, et al. Alteration of plasma glutamate and glutamine levels in children with high-functioning autism. *PLoS One.* 2011;6(10):e25340.
70. Saha S, Saha T, Sinha S, Rajamma U, Mukhopadhyay K. Autistic traits and components of the folate metabolic system: an explorative analysis in the eastern Indian ASD subjects (dagger). *Nutr Neurosci.* 2019:1-8.
71. Sweetman DU, O'Donnell SM, Lalor A, Grant T, Greaney H. Zinc and vitamin A deficiency in a cohort of children with autism spectrum disorder. *Child Care Health Dev.* 2019;45(3):380-386.
72. Yektas C, Alpay M, Tufan AE. Comparison of serum B12, folate and homocysteine concentrations in children with autism spectrum disorder or attention deficit hyperactivity disorder and healthy controls. *Neuropsychiatr Dis Treat.* 2019; 15:2213-2219.
73. Afrazeh M, Saedisar S, Khakzad MR, Hojati M (2015), Measurement of Serum Superoxide Dismutase and Its Relevance to Disease Intensity Autistic Children. *Maedica (Buchar)* 10:315-318.
74. Altun H, Kurutas EB, Sahin N, Gungor O, Findikli E (2018), The Levels of Vitamin D, Vitamin D Receptor, Homocysteine and Complex B Vitamin in Children with Autism Spectrum Disorders. *Clin Psychopharmacol Neurosci* 16:383-390.
75. Bicikova M, Macova L, Ostatnikova D, Hanzlikova L (2019), Vitamin D in autistic children and healthy controls. *Physiol Res* 68:317-320.
76. Chen J, Xin K, Wei J, Zhang K, Xiao H (2016), Lower maternal serum 25(OH) D in first trimester associated with higher autism risk in Chinese offspring. *J Psychosom Res* 89:98-101.
77. Cheng B, Zhu J, Yang T, Guo M, Lai X, Li Q, Chen J, Li T (2020), Vitamin A deficiency increases the risk of gastrointestinal comorbidity and exacerbates core symptoms in children with autism spectrum disorder. *Pediatr Res.*
78. Ghodsi R, Kheirouri S (2019), Positive Association Between Plasma Levels of Advanced Glycation and Precursor of Lipoxidation end Products with Gastrointestinal Problems in Children with Autism. *Curr Pediatr Rev* 15:184-190.
79. Guo M, Li L, Zhang Q, Chen L, Dai Y, Liu L, Feng J, Cai X, et al. (2018), Vitamin and mineral status of children with autism spectrum disorder in Hainan Province of China: associations with symptoms. *Nutr Neurosci*:1-8.
80. Guo M, Zhu J, Yang T, Lai X, Lei Y, Chen J, Li T (2019), Vitamin A and vitamin D deficiencies exacerbate symptoms in children with autism spectrum disorders. *Nutr Neurosci* 22:637-647.
81. Hodgson NW, Waly MI, Al-Farsi YM, Al-Sharbati MM, Al-Farsi O, Ali A, Ouhtit A, Zang T, et al. (2014), Decreased glutathione and elevated hair mercury levels are associated with nutritional deficiency-based autism in Oman. *Exp Biol Med (Maywood)* 239:697-706.

82. James SJ, Melnyk S, Fuchs G, Reid T, Jernigan S, Pavliv O, Hubanks A, Gaylor DW (2009), Efficacy of methylcobalamin and folinic acid treatment on glutathione redox status in children with autism. *Am J Clin Nutr* 89:425-430.
83. Main PA, Thomas P, Angley MT, Young R, Esterman A, King CE, Fenech MF (2015), Lack of evidence for genomic instability in autistic children as measured by the cytokinesis-block micronucleus cytome assay. *Autism Res* 8:94-104.
84. Ning J, Xu L, Shen CQ, Zhang YY, Zhao Q (2019), Increased serum levels of macrophage migration inhibitory factor in autism spectrum disorders. *Neurotoxicology* 71:1-5.
85. Russo AJ, Devito R (2011), Analysis of Copper and Zinc Plasma Concentration and the Efficacy of Zinc Therapy in Individuals with Asperger's Syndrome, Pervasive Developmental Disorder Not Otherwise Specified (PDD-NOS) and Autism. *Biomark Insights* 6:127-133.
86. Saad K, Abdel-Rahman AA, Elserogy YM, Al-Atram AA, Cannell JJ, Bjorklund G, Abdel-Reheim MK, Othman HA, et al. (2016), Vitamin D status in autism spectrum disorders and the efficacy of vitamin D supplementation in autistic children. *Nutr Neurosci* 19:346-351.
87. Sun C, Zou M, Zhao D, Xia W, Wu L (2016), Efficacy of Folic Acid Supplementation in Autistic Children Participating in Structured Teaching: An Open-Label Trial. *Nutrients* 8.
